# Classification of fungal and bacterial lytic polysaccharide monooxygenases

**DOI:** 10.1186/s12864-015-1601-6

**Published:** 2015-05-09

**Authors:** Peter K Busk, Lene Lange

**Affiliations:** Department of Chemistry and Bioscience, Aalborg University and Barentzymes A/S, A.C. Meyers Vænge 15, 2450, Copenhagen, SV Denmark

**Keywords:** Lytic polysaccharide monooxygenases, Subfamilies, Sequence analysis, Peptide pattern recognition, Genomic annotation

## Abstract

**Background:**

Lytic polysaccharide monooxygenases are important enzymes for the decomposition of recalcitrant biological macromolecules such as plant cell wall and chitin polymers. These enzymes were originally designated glycoside hydrolase family 61 and carbohydrate-binding module family 33 but are now classified as auxiliary activities 9, 10 and 11 in the CAZy database. To obtain a systematic analysis of the divergent families of lytic polysaccharide monooxygenases we used Peptide Pattern Recognition to divide 5396 protein sequences resembling enzymes from families AA9 (1828 proteins), AA10 (2799 proteins) and AA11 (769 proteins) into subfamilies.

**Results:**

The results showed that the lytic polysaccharide monooxygenases have two conserved regions identified by conserved peptides specific for each AA family. The peptides were used for *in silico* PCR discovery of the lytic polysaccharide monooxygenases in 79 fungal and 95 bacterial genomes. The bacterial genomes encoded 0 – 7 AA10s (average 0.6). No AA9 or AA11 were found in the bacteria. The fungal genomes encoded 0 – 40 AA9s (average 7) and 0 – 15 AA11s (average 2) and two of the fungi possessed a gene encoding a putative AA10. The AA9s were mainly found in plant cell wall-degrading asco*-* and basidiomycetes in agreement with the described role of AA9 enzymes. In contrast, the AA11 proteins were found in 36 of the 39 ascomycetes and in only two of the 32 basidiomycetes and their abundance did not correlate to the degradation of cellulose and hemicellulose.

**Conclusions:**

These results provides an overview of the sequence characteristics and occurrence of the divergent AA9, AA10 and AA11 families and pave the way for systematic investigations of the of lytic polysaccharide monooxygenases and for structure-function studies of these enzymes.

**Electronic supplementary material:**

The online version of this article (doi:10.1186/s12864-015-1601-6) contains supplementary material, which is available to authorized users.

## Background

Copper-dependent lytic polysaccharide monooxygenases (LPMOs) are important enzymes for degradation of biological macromolecules such as plant cell wall and chitin polymers [1-6]. The LPMOs are metalloenzymes that oxidize the glycosidic bonds in cellulose [7-9], hemicellulose [1] and chitin [6]. The catalysis involves binding of an active oxygen molecule to the copper atom [5,7,8,10] and interaction of a large surface of the LPMO with several chains of the crystalline polysaccharide substrate [11-13]. It was hypothesized that LPMOs could only act on crystalline substrates but recently it was found that some LPMOs oxidize soluble short-chain polysaccharides hence expanding the known LPMO substrates to soluble polymers [10].

The LPMOs were originally classified as glycoside hydrolases (GHs) and carbohydrate-binding modules (CBMs) but are now placed in the auxiliary activity families AA9, AA10 and AA11 in the CAZy database [14].

Family AA9 include fungal enzymes formerly classified in the GH61 family. Their classification as GHs was based on the report that one of the GH61s has weak endocellulase activity [15]. Furthermore, certain AA9 family members enhance enzymatic degradation of cellulose [3] and are important industrial enzymes for conversion of lignocellulotic biomass into soluble sugars.

The enzymatically characterized AA9 proteins oxidise the glycosidic bonds in cellulose [7-9] but less than ten of the more than 200 AA9 proteins in the CAZy database (www.cazy.org) have been investigated. Furthermore, based on the large sequence diversity of the AA9 proteins it has been suggested that some AA9s may oxidize other substrates than cellulose [12,16,17]. This hypothesis is supported by a recent report of an AA9 protein that degrades hemicellulose [1] and different substrates induce expression of different AA9 genes [18-20]. Moreover, some fungi possess more than 30 different AA9-encoding genes in their genomes [3], which seems to be an excessive number of LPMOs for degradation of just one substrate.

In this context it is interesting that some of the bacterial LPMOs in the AA10 family degrade chitin whereas other degrade cellulose [2,6,21,22]. In addition, the substrate for the only characterized enzymes of the other type of fungal LPMOs, the AA11 proteins, is chitin [23]. The AA11 LPMOs were originally classified as a subfamily of the GH61 proteins [24] but later renamed as AA11. However, only a few of these proteins have been annotated and only a single one is functionally characterized [23].

Peptide Pattern Recognition (PPR) is a new approach for sequence analysis. It was used to classify 743 GH61-like proteins and pinpoint conserved amino acid residues in their sequences [24]. Moreover, PPR was used to annotate all GHs and LPMOs in 40 fungal genomes [25]. However, only LPMOs similar to the sequences found in CAZy were annotated. To further investigate the large number of fungal LPMOs of the AA9 and AA11 families and the high sequence diversity we have used PPR to divide 833 LPMOs found in CAZy and additional 4456 LPMO-like proteins found by BLAST search in GenBank into subfamilies. Each of the three families AA9, AA10 and AA11 were characterized by a non-overlapping set of conserved peptides that mapped to similar regions in the LPMO proteins. The conserved sequences were used to annotate the LPMOs in 79 fungal and 95 bacterial genomes. AA9 and AA11 were exclusively found in fungal genomes whereas AA10s were found in bacteria and a single AA10-encoding gene was found in each of two fungi. Furthermore, AA9s were more abundant in plant cell wall-degrading fungi in agreement with that the substrates for AA9 enzymes are cellulose and hemicellulose. In contrast, AA11-encoding genes were found in all the ascomycetes examined, except for three saccharomycetes with different life styles, but only in a few of the white and brown rot basidiomycetes and the number of genes did not correlate to the degradation of cellulose and hemicellulose.

## Results

Division of large families of CAZyme proteins like the GH13 and GH5 into subfamilies has proven to be an important tool for characterization of the families [26,27]. To obtain subfamilies of the LPMOs 248 AA9, 537 AA10 and 48 AA11 unique protein sequences were downloaded from GenBank. Furthermore, 36 GH61 subfamily 7 proteins [24] were pooled with the AA11 sequences yielding a total of 80 unique AA11/GH61 subfamily 7 sequences after removal of duplicates. PPR analysis of the proteins after removal of duplicates and of irrelevant protein domains generated 12 subfamilies of AA9 proteins, 20 subfamilies of AA10 proteins and 3 subfamilies of AA11 proteins (Additional file 1).

Due to the careful curation of sequences in CAZy [28,29] this database may not contain all sequences relevant for sequence analysis of the LPMOs [24]. Therefore, we used BLASTp search to find the 1000 sequences with the highest sequence similarity to the sequence with highest score in each of the LPMO subfamilies. These sequences were curated by removal of duplicates and irrelevant proteins domains and all sequences recognized as AA9 by PPR from the list of AA11-like sequences and vice versa (see Methods). A procedure of removing irrelevant protein domains rather than relying on positive identification of LPMO-relevant domains by CDD search was used to include putative LPMOs that might not contain a domain recognized by the CDD (see Methods, Sequence Analysis). Although this approach increases the risk of including non-LPMO proteins in the analysis we have previously found that PPR analysis separates unrelated proteins into different subfamilies [24,30]. Hence, these subfamilies may be removed after PPR analysis by manual curation.

The expanded LPMO families consisted of 1798 AA9-like proteins (AA9exp), 2799 AA10-like proteins (AA10exp) and 692 AA11-like proteins (AA11exp), respectively. This represents an additional 1550 AA9-like proteins, 2262 AA10-like proteins and 612 AA11-like proteins.

**Figure 1:**
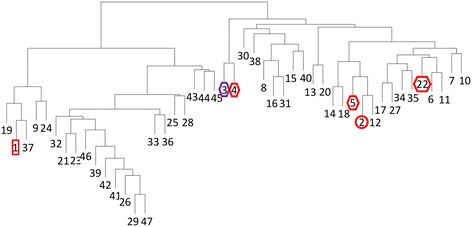
Cluster analysis of the 47 AA9exp subfamilies. Cluster analysis based on the number of shared hexapeptides between each subfamily. The subfamilies that included AA9 proteins designated as enzymatically characterized in the CAZy database are indicated. Subfamilies containing C1-oxidizing LPMOs are indicated with a circle and C4-oxidizing LPMOs with a square. Subfamilies containing LPMOs that are both C1- and C4- oxidizing or with unknow mechanism are indicated with a diamond. The symbol around subfamilies with cellulose-oxidizing members is red and the symbol around subfamily 3 which contains a cellulose- and hemicellulose-oxidizing member is purple. Cluster analysis was performed as described in Methods.

PPR divided the AA9exp proteins into 47 subfamilies (Additional file 1). Comparison of the conserved peptides between subfamilies showed that the AA9exp subfamilies shared from 0 to 10 conserved peptides (Figure 1 and Additional file 2). Interestingly, of the eight enzymatically characterized AA9s three are classified in the related subfamilies 3 and 4, two in subfamily 1, two in the related subfamilies 2 and 5 and the last one in subfamily 22 (Figure 1). Thus no AA9s from a large group of subfamilies (subfamilies 21, 23, 25, 26, 28, 29, 32, 33, 36, 39 and 41 - 47) have been characterized although they are distantly related to the other subfamilies.

**Figure 2:**
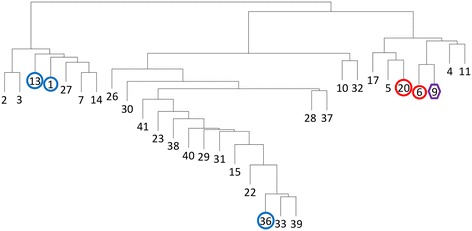
Cluster analysis of the 31 AA10exp subfamilies. Cluster analysis based on the number of shared hexapeptides between each subfamily. The subfamilies that included AA10 proteins designated as enzymatically characterized in the CAZy database are indicated with the same symbols as in Figure 1. The symbol around subfamilies with cellulose-oxidizing members is red and the symbol around subfamilies with chitin-oxidizing members is blue. Subfamily 9 is indicated in purple as it contains members oxidizing both cellulose and chitin Cluster analysis was performed as described in Methods.

To make sure that all relevant AA10-like proteins were included in the expanded AA10 family less stringent criteria were used to exclude protein domains from the sequences of the AA10-like proteins (see Methods). As a result of this 13 of the 44 subfamilies of the AA10exp proteins generated by PPR contained proteins that were identified as not being LPMOs, not including a GH61 or chitin-binding domain as accessed by CDD search. After removal of these irrelevant subfamilies, there were 31 subfamilies of AA10 proteins (Additional file 1) that shared 0 – 8 conserved peptides (Additional file 2). The enzymatically characterized AA10s were classified to subfamilies that are relatively well distributed in the cluster analysis (Figure 2). Interestingly, the three AA10s that oxidize cellulose belong to subfamilies 6, 9 and 20 that cluster close together. Thus the AA10 subfamily division does correlate to enzyme substrate to a certain extent although subfamily 9 also contains a chitin-degrading AA10.

**Figure 3:**
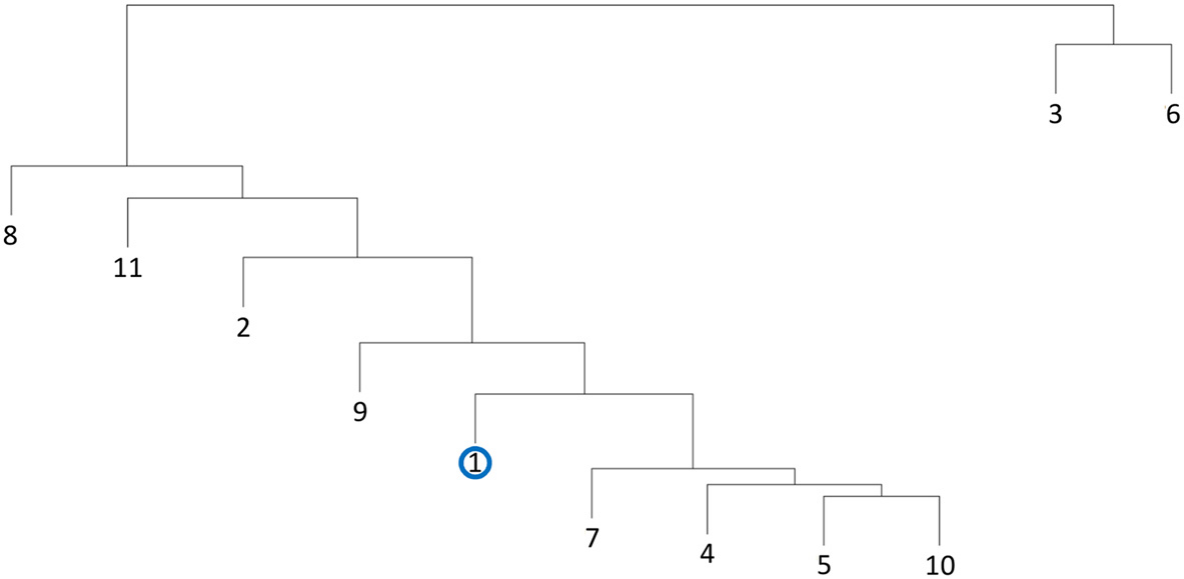
Cluster analysis of the 11 AA11exp subfamilies. Cluster analysis based on the number of shared hexapeptides between each subfamily. Subfamily 1 that included the AA11 protein designated as enzymatically characterized in the CAZy database is indicated with the same symbol as in Figure 1. The symbol is blue to indicate the the characterized enzyme oxidizes chitin. Cluster analysis was performed as described in Methods.

PPR analysis divided the AA11exp proteins into 11 subfamilies (Additional file 1). Seven of the subfamilies were closely related and share up to 13 conserved peptides (Additional file 2). The only characterized AA11 is classified to one of these subfamilies. (Figure 3). The last four subfamilies only shared few peptides with the other subfamilies (Additional file 2).

**Figure 4:**
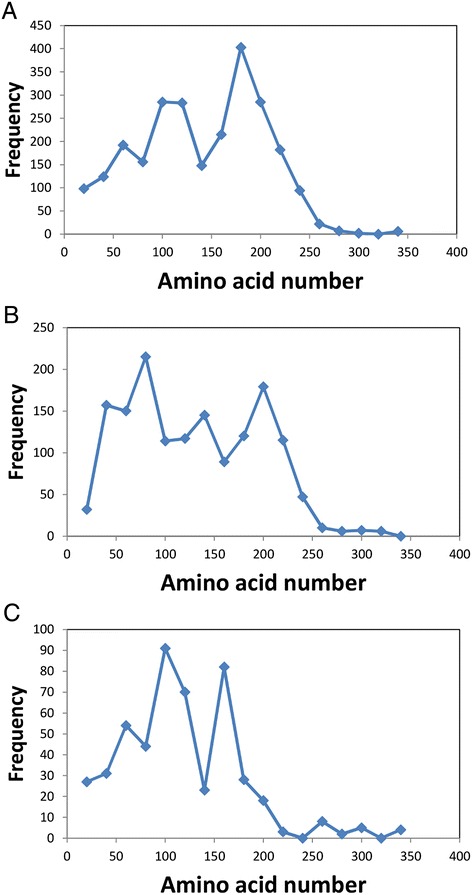
Distribution of the conserved hexapeptides in the LPMO sequences. The distribution of hexapeptides for each subfamily was calculated as the number of hexapeptides mapping to each 20 amino acids interval as described in Methods. The accumulated hexapeptide frequency (vertical axis) was calculated as the sum of the distribution of all the subfamilies in each 20 amino acids interval. The horizontal axis designates the amino acid intervals. **A**: AA9exp. **B**: AA10exp. **C**: AA11exp.

No peptides were shared between any of the AA9exp subfamilies and the AA10exp subfamilies or the AA11exp subfamilies or between any of the AA10exp subfamilies and the AA11exp subfamilies. Hence, the three families of LPMOs can be clearly separated based on their conserved peptides. Nevertheless, the distribution of conserved peptides on the LPMOs identifies a highly conserved region around amino acids 160 – 180 (AA9), 180 – 200 (AA10) and 140 – 160 (AA11) (Figure 4). Furthermore, the AA9s and AA11s have another conserved region around amino acids 80 – 120. This conserved region is located a little differently in the AA10s around amino acids 60 – 80 (Figure 4). Very few conserved peptides were identified after amino acid 250 in all three families.

**Figure 5:**
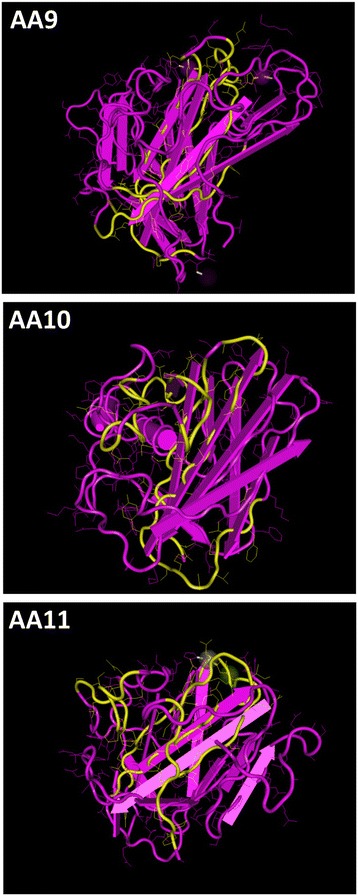
Mapping of conserved peptides in AA9, AA10 and AA11 on 3D proteins structures. Conserved peptides from AA9 subfamily 1 was mapped onto the structure (PDB ID: 3ZUD) of *T. aurantiacus* AA9 [8] belonging to subfamily 1. Conserved peptides from AA10 subfamily 1 was mapped onto the structure (PDB ID: 2YOW) of *B. amyloliquefaciens* AA10 [31] belonging to AA10 subfamily 1. Conserved peptides from AA11 subfamily 1 was mapped onto the structure (PDB ID: 4MAH) of *A. oryzae* AA11 [23] belonging to AA11 subfamily 1. The regions including conserved peptides are indicated in yellow. For *T. aurantiacus* AA9 [8] the conserved regions are amino acids 102-121, 131-139, 171-189 and 192-203. For *B. amyloliquefaciens* AA10 [31] the conserved regions are amino acids 60-75, 80-88, 171-192 and 194-204. For *A. oryzae* AA11 [23] the conserved regions are amino acids 42-52, 75-88, 143-165 and 190-197. Amino acid side chains are shown as thin strings. Alpha-helixes are indicated as tubes and beta-sheets are indicated by flat arrows.

Mapping of the conserved residues onto the 3D-structure of an LPMO belonging to each family showed that the conserved region extends through the center of the proteins and protrudes on both sides of the structure (Figure 5). Hence a large part of the conserved region is buried inside the proteins as previously reported [24]. The 3D structures used were LPMO proteins from *Thermoascus aurantiacus* [8] belonging to AA9 subfamily 1, *Bacillus amyloliquefaciens* [31] belonging to AA10 subfamily 1 and *Aspergillus oryzae* [23] belonging to AA11 subfamily 1.

We have previously shown that the conserved peptides identified by PPR in LPMOs are useful for design of degenerate primers for PCR amplification of related genes [24]. Moreover, by searching in proteins sequences for homology to peptide patterns (implemented as Hotpep), it can be investigated whether a protein sequence contains a sufficient number of conserved peptides from a specific PPR-generated LPMO subfamily to be identified as an LPMO and member of this subfamily [25]. This is a kind of *in silico* PCR with degenerated primers.

To test whether the conserved peptides can be used not only to annotate LPMOs but also to distinguish between the three different LPMO families we used Hotpep to annotate the AA9, AA10 and AA11 proteins in CAZy with the conserved peptides for the LPMO families. Hotpep annotated between 96 and 100% of the AA9, AA10 and AA11 proteins to the correct family and none of the proteins were falsely annotated (Table 1). For the AA9exp, AA10exp and AA11exp the annotation rate was lower. However, most of the unannotated proteins were suspected not to be LPMOs. Only 3% of the unannotated AA9exp sequences, 4% of the unannotated AA10exp sequences and 2% of the unannotated AA11exp sequences included a GH61 (AA9exp and AA11exp) or chitin-binding domain (AA10exp and AA11exp) as accessed by CDD search. After removal of the proteins without a domain clearly associated to LPMOs from the sequence list, the annotation rate increased to 96% (AA9exp), 94% (AA10exp) and 98% (AA11exp). These data indicate that the peptide patterns generated for the three LPMO families were able to recognize the vast majority of the LPMOs without false annotation of unrelated sequences.

**Table 1:** **Percent of LPMO-encoding genes annotated with the conserved peptides**

**Pep list**	**AA9exp**	**AA10exp**	**AA11exp**
AA9 proteins	98	0	0
AA10 proteins	0	96	0
AA11 proteins	0	0	100
AA9exp proteins	91	0	0
AA10exp proteins	0	75	0
AA11exp proteins	0	0	73

To investigate the potential of different fungi for expression of LPMOs we used Hotpep [25] to mine 79 fungal genomes for LPMO-encoding genes. This was done by dividing the contigs of each genome into 2000 nucleotides long fragments and search for conserved peptides from each subfamily of the AA9exp, AA10exp and AA11exp families in all six reading frames. All reading frames that satisfied the threshold conditions (see Methods) were considered as hits.

For 39 of the fungal genomes the LPMOs have previously been annotated based on similarity to the LPMOs found in CAZy [25]. This analysis identified 284 LPMOs in the 39 genomes. However, by using the expanded LPMO families, a total of 373 LPMOs were found in the same 39 genomes.

**Figure 6:**
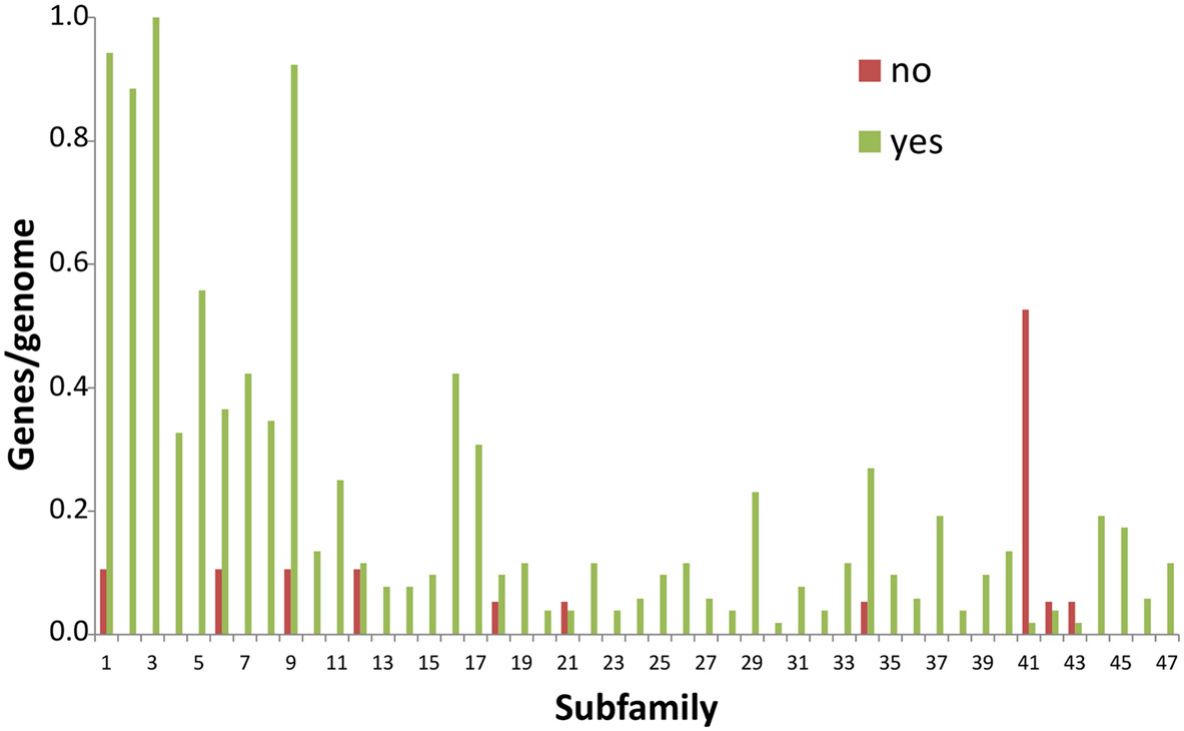
Distribution of genes for each AA9 subfamily in the fungal genomes. The number of genes encoding proteins belonging to each AA9 subfamily was counted for each genome. Plant cell wall-degraders are indicated by “yes” and non-degraders are indicated by “no”. The classification of fungi as plant cell wall-degraders or non-degraders is described in “Methods”.

For all of the 79 fungal genomes AA9-encoding genes were found in the ascomycetes and the basidiomycetes but not in the three other fungal phyla whereas AA11-encoding genes were only found in the ascomycetes and in three of the basidiomycetes (Table 2 and Additional file 3). The fungi were designated as plant cell wall-degrading and non-degraders based on whether they have been reported to degrade major components (cellulose, hemicellulose, lignin) of natural plant cell wall material as previously described [25]. According to this definition, the brown and white rot basidiomycetes and saprophytic fungi are designated as lignocellulose degraders whereas zygomycetes and chytridiomycetes that are able to degrade pure cellulose in the laboratory but have not been reported to decompose natural lignocellulotic compounds in nature are designated as non-degraders [32-37]. The plant cell wall-degrading fungi of all phyla had an average of ten AA9-encoding genes although there were large variations from a few genes in *Talaromyces* and *Trichoderma* spp. and brown rot fungi to 40 AA9 genes in *Chaetomium globosum*. There were more AA9 belonging to some subfamilies, most notably subfamilies 3, 5 and 17, in the plant cell wall-degraders than in the other ascomycetes and basidiomycetes (Figure 6 and Additional file 4). Moreover, no AA9 LPMOs were found in any of the eight dermatophytes *Arthroderma gypseum, Arthroderma otae, Candida albicans, Coccidioides immitis, Coccidioides posadasii, Trichophyton rubrum, Trichophyton tonsurans* and *Trichophyton verrucosum*or in the two insect pathogens *Cordyceps militaris* and *Metarhizium anisopliae*.

**Table 2:** **Average number of LPMO-encoding genes in 79 fungal and 95 bacterial genomes**

	**AA9exp**	**AA10exp**	**AA11exp**
Ascomycetes	7.4	0.0	3.7
Basidiomycetes	8.0	0.1	0.3
Chytridiomycetes	0.0	0.0	0.0
Zygomycetes	0.0	0.0	0.0
Blastochladiomycetes	0.0	0.0	0.0
Non-degrading fungi	1.5	0.0	1.9
Cell-wall degrading fungi	10	0.0	2.0
Eubacteria	0.0	0.6	0.0

**Figure 7:**
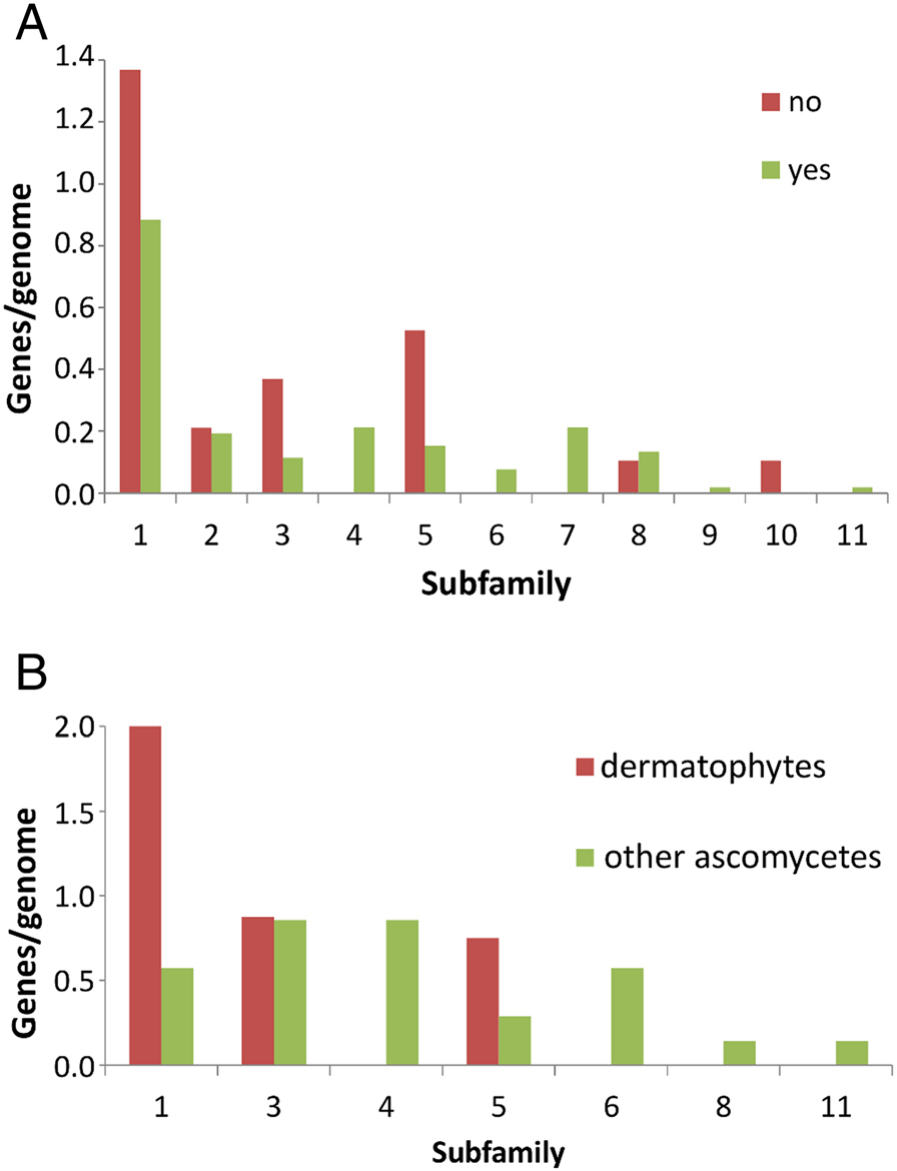
Distribution of genes for each AA11 subfamily in the genomes of ascomycetes. The number of genes encoding proteins belonging to each AA9 subfamily was counted for each genome. **A**. Plant cell wall-degraders are indicated by “yes” and non-degraders are indicated by “no”. **B**. Dermatophytes are indicated by “dermatophyte” and non-invaders are indicated by “other ascomycete”. The classification of fungi as plant cell wall-degraders, non-degraders, dermatophytes and other ascomycetes is described in “Methods” and in Additional file 4.

The number of AA11-encoding genes in the fungal genomes did not correlate to the capacity of the fungi to degrade cellulose and hemicellulose (Table 2 and Additional file 3). As the AA11-encoding genes were only found in the genomes of the ascomycetes we investigated whether there was a correlation between AA11 subfamilies and the life style of the acomycetes. Subfamilies 4- and 7-encoding genes were found in more than half of the plant cell wall degraders but not in any of the non-degraders (Figure 7). Although these subfamilies clustered close to subfamily 5 (Figure 3) that was more abundant in non-degraders than in plant cell wall-degraders the result suggests that AA11 LPMOs belonging to subfamilies 4 and 7 may be related to plant cell wall degradation. Next, the subfamily distribution of the AA11-encoding genes in the dermatophytes was compared to the genes in the seven non-dermatophytic ascomycetes *Botryotinia fuckeliana, Ceratocystis fimbriata, Saccharomyces cerevisiae, Uncinocarpus reesii, Wickerhamomyces anomalus, Daldinia eschscholzii* and *Bipolaris maydis*. This showed that that the dermatophytic ascomycetes had on average more than three times as many AA11 subfamily 1-encoding genes than the non-dermatophytic ascomycetes (Figure 7). Therefore, some AA11 LPMOs may be involved in degradation of the keratinized skin components that dermatophytes use as nutrient [38]. On the other hand, AA11 subfamily 4 was found in the genomes of the endophytic ascomycetes *D. eschscholzii* [39] and *Ascocoryne sarcoides* [40] and the white rot-causing basidiomycete *Schizophyllum commune* [41] (Additional file 4).

Six AA10-encoding genes were predicted in the 79 fungal genomes but upon closer examination by BLASTp search of the encoded amino acid sequenced it was found that only two of these genes from the litter-decomposing basidiomycete *Galerina marginata* and the smut *Ustilago maydis,* which is a maize pathogen, encode putative AA10 proteins. The closest BLAST hit to the gene from *G. marginata* was a bacterial gene from *Streptomyces rimosus* (E value = 3×10^−51^) whereas the closest hits to the gene from *U. maydis* were seven genes from Basidiomycota (*Pseudozyma hubeiensis, Ustilago hordei, Sporisorium reilianum, Pseudozyma antarctica, Pseudozyma aphidis, Pseudozyma flocculosa* and *Pseudozyma brasiliensis* (E values = 8×10^−128^ - 2×10^−68^) but also genes from *Streptomyces* sp. were closely related (E value = 4×10^−55^).

To investigate whether the AA9 and AA11 are found in bacteria we used Hotpep to mine 95 bacterial genomes for LPMOs. However, no AA9- or AA11-encoding genes were found in the bacteria, which had from 0 to 7 AA10-encoding genes (Table 2 and Additional file 5). The finding of 545 AA9s, 156 AA11s and only 2 AA10s in the 79 fungal genomes and 61 AA10s but no AA9s or AA11s in the bacterial genomes supports the notion that AA9 and AA11 are eukaryotic LPMOs whereas AA10 are bacterial.

## Discussion

The reclassification of the LPMOs as auxiliary activities based on their enzymatic and structural similarities is an important step towards a systematic analysis of these enzymes and their substrate preferences [14]. However, each of the three families of LPMOs contains a large number of very divergent sequences and only a few of these have been enzymatically characterized. Hence, the further division of the LPMOs into PPR-generated subfamilies of proteins with related sequences provides a short cut to a systematic characterization of the LPMOs. To obtain as comprehensive sequence information about the LPMOs as possible we included not only the AA9, AA10 and AA11 sequences listed in the CAZy database in the PPR analysis but also a wide selection of LPMO-like sequences found by similarity search in GenBank. We have previously used this approach to analyze the AA9 proteins, which lead to the discovery of the AA11 proteins that were classified as GH61 subfamily 7 [24]. In the present work the expanded LPMO families were divided into 47 AA9, 31 AA10 and 11 AA11 subfamilies compared to only 12 AA9, 20 AA10 and 3 AA11 subfamilies when only LPMO sequences from the CAZy database were included. Thus, the expansion leads to a more comprehensive characterization of the families and is easily handled by the PPR algorithm. One drawback is that a number of proteins that are not true LPMOs were included in the expanded sequence pools. This complication was reduced by removing most domains not relevant for the LPMO catalytic activity from the sequences before PPR analysis. Furthermore, after PPR analysis each subfamily was examined by CDD and BLAST search [42,43] and inspected manually to remove all subfamilies that do not include LPMOs. We have previously found that grouping proteins with PPR leads to exclusion of unrelated proteins [24,30]. Thus, any non-LPMO sequence that might have sneaked in to the sequence pool was excluded from the subfamilies of LPMOs. This approach made it possible to annotate 31% more LPMOs based on the expanded LPMO lists in the 39 fungal genomes that have previously been investigated and annotated based only on the LPMOs found in CAZy [25].

For GH families including proteins with different functions PPR divides the proteins into subfamilies that correlate largely to the functions of the proteins [24]. An important purpose of the present analysis was to correlate sequence information for the LPMOs to functional data. As the enzymatically characterized AA9 proteins were classified to only six of the 47 subfamilies there is a host of AA9s with very different sequences that remain to be characterized. Comparison of AA9s from plant cell wall degrading asco- and basidiomycetes to AA9s in non-degrading asco- and basidiomycetes showed which of these subfamilies may be associated to degradation of lignocellulose. However, LPMOs with high ability to enhance lignocellulose degradation may belong to different subfamilies as exemplified by the *T. aurantiacus* LPMO and the *Thielavia terrestris* LPMO [3] classified in subfamily 1 and subfamily 2 that were very distant in the cluster analysis of the AA9 subfamilies. The catalytic activities of the LPMOs in subfamily 1 have been reported as endoglucanase for the enzyme from *Trichoderma reesei* [15] and monooxygenase yielding C1- and C4 oxidized products for the enzyme from *Podospora anserina* [44]. Three AA9 enzymes from *Neurospora crassa* fall into the closely related subfamilies 3 and 4 but represent different catalytic mechanisms as the two enzymes in subfamily 3 are C1-oxidizing and C4-oxidizing and the enzyme in subfamily 4 is both C1- and C4-oxidizing [5,7,10,45]. Likewise, the enzymes in the related subfamilies 2 and 5 are a C1-oxidising LPMO from *N. crassa* and a C1- and C4-oxidizing LPMO from *Phanerochaete chrysosporium* [5,7,9,13,45]. Hence, there does not seem to be a clear correlation between the catalytic mechanisms of the AA9 LPMOs and their subfamily annotation. The classification of a hemicellulose-oxidizing LPMO [1] into subfamily 3 together with a cellulose-oxidizing LPMO [5,7] does not point to a clear division of the LPMOs based on substrate recognition. However, there is limited knowledge about the substrate specificity of the LPMOs. Hence, more LPMOs need to be characterized to reach a definite conclusion. Interestingly, many genomes of fungi degrading plant cell wall material possess several genes encoding betaglucosidases, endoglucanases and AA9-type LPMOs indicating that degradation of different, complex substrates may require different enzyme with the same activity [25]. This notion points to that a comprehensive understanding of the properties of different LPMOs requires studies on complex natural substrates.

The AA10 family proteins have been described to oxidize two substrates, chitin and cellulose, that are built from different monomer carbohydrates. Interestingly, the cellulose- oxidizing AA10s [2,11,22] are found in the closely related subfamilies 6, 9 and 20 that are far from the subfamilies 1, 13 and 36 that contain only chitin-oxidizing AA10s [6,11,21,46,47]. Although the characterized AA10 protein from *Thermobifida fusca* in subfamily 9 is able to oxidize both cellulose and chitin [22] this separation of the LPMOs in the AA10 family according to substrate indicates that the subfamily classification may be used to predict the putative substrate of uncharacterized LPMOs. However, a confirmation of this possibility will await biochemical characterization of more LPMOs.

In contrast to the AA9 LPMOs, the number of AA11-encoding genes did not correlate to the degradation of cellulose and hemicellulose but rather to the fungal taxonomy as these genes were found in 36 of the 39 ascomycetes and in only four of the 32 basidiomycetes: The non-cellulose-degrading *Cryptococcus neoformans* and *Tremella mesenterica,* the white rot fungus *Ceriporiopsis subvermispora* and the white rot-causing fungus *S. commune.* The putative amino acid sequences encoded by the genes from *C. neoformans* and *T. mesenterica* share 83 of 150 amino acids and 76 of 160 amino acids, respectively, with a gene from the dermatophyte *Blastomyces dermatitidis*. The seven genes from *S. commune* are closely related in groups of two and five genes and appear to originate from two different genes. The sequence from *C. vermispora* is only 100 amino acids long and does not have any significant hits in BLASTp search. Interestingly, the comparison of the occurrence of AA11 subfamilies showed that dermatophytes have more than three times as many AA11 subfamily 1 encoding-genes than other ascomycetes. This result suggests that skin may contain a substrate for AA11 subfamily 1. Dermatophyte invasion in immunocompetent individuals is usually restricted to the keratinized, non-living material and involves the expression of a host of keratinases [38,48,49]. It is likely that the AA11 takes part in this degradation and may attack keratin or another macromolecule of the extracellular matrix. The only enzymatically characterized AA11 protein from *A. oryzae* is a chitinase [23], which is classified in subfamily 1. A number of genes encoding proteins with a chitin-binding LysM domain have been identified in the genomes of dermatophytes [49]. The dermatophyte genomes do also contain chitinase-encoding genes and it is likely that some of their LysM-domain containing proteins are involved in chitin degradation [49]. However, some of the LysM-encoding genes of *T. rubrum* are specifically expressed when this dermatophyte grows on keratin thus suggesting a link between degradation of keratin and apparent chitin-binding proteins [50]. In analogy with the LysM-containing proteins the AA11 proteins from subfamily 1 may be used by the dermatophytes for growth on chitin but it is also possible that they have a role in growth on keratin.

In the context of industrial use of LPMOs for degradation of plant cell wall materials and productions of biofuels such as ethanol [1,3] the present results indicate the search for new LPMO enzymes should focus on the AA9 family and possibly on the AA10 subfamilies that contain cellulose-degrading enzymes and on related subfamilies.

The two basidiomycetes *C. neoformans* and *S. commune* that posses AA11-encoding genes are known to cause human infections [51-53] and it would be interesting to investigate whether their AA11 genes are induced during infection. On the other hand, no reports link the basidiomycete *T. mesenterica* that has an AA11-encoding gene, to human infections but this fungus is a fungal parasite [54] and could use the AA11 protein for degradation of the chitin-containing cell wall of its host.

Overall, the genome analysis is consistent with that AA9 and AA11 are eukaryotic LPMOs whereas AA10 are bacterial. Nevertheless, the genomes of the litter-decomposing basidiomycete *G. marginata* and the smut *U. maydis* encoded AA10-like proteins. These genes were closely related to bacterial LPMOs suggesting that *G. marginata* and *U. maydis* acquired their AA10 genes through horizontal gene transfer [55]. For example, in its yeast form *U. maydis* interacts with bacteria in the phyllosphere, which contains a large variety of bacteria and fungi [56,57]. The considerably larger number of LPMOs that were generally found in the fungal genomes compared to bacterial genomes is consistent with the notion that eukaryotic genomes are larger, often redundant and contains more genes than eubacterial genomes [58].

Comparison of the distribution of the conserved peptides on the LPMO sequences indicated two conserved regions in accordance with previous findings for the GH61 family [24]. One of the conserved regions is consistent with the substrate binding site of the LPMOs and the conserved surface residues in each subfamily have been identified and may be involved in recognition of different substrates [24]. In the present work we found that also the AA10 family had a conserved region around amino acids 180 - 200 but a slightly different structure of conserved peptides towards the N-terminal than the AA9 and AA11 families.

We have previously shown that PPR can be used for dividing 743 AA9 and AA11 proteins into subfamilies [24]. In the present work PPR demonstrated its ability to subdivide 5396 LPMOs. This subdivision could be used to annotate the LPMOs in fungal and bacterial genomes. It is also possible to annotate CAZymes by the use of a gene prediction algorithm followed by mapping of conserved domains [43] or directly through the CAZy database [29]. However, by using PPR the training set can be expanded beyond the sequences found in CAZy, which is a useful feature when an exhaustive analysis is required. This feature is also important for analysis of protein families that are not classified in high-quality databases such as CAZy.

## Conclusions

The LPMOs are important enzymes for microbiological degradation of cellulose, chitin and related poly- and oligoshaccharides [1-6]. Nevertheless, only a few of these enzymes have been characterized. The classification of the LPMOs into the three protein families AA9, AA10 and AA11 is an important step towards understanding the divergence of this large group of very divergent sequences [23]. In the present work we have collected the LPMOs and available LPMO-like sequences from public databases and arranged them into subfamilies. This analysis showed that the functionally characterized LPMOs do only represent a small part of the natural sequence variation of these enzymes. Moreover, it pinpointed the subfamilies of LPMOs that have not been functionally characterized and thus have the highest probability of having different properties than the already described LPMOs. In this context it is relevant that the AA10 enzymes that oxidize cellulose belong to three closely related subfamilies. Moreover, some AA11 subfamilies were mainly found in dermatophytic ascomycetes pointing to a putative function of these enzymes in keratinolysis. In contrast to the substrate preference of the AA10 enzymes there does not seem to be a correlation between the subfamilies and a C1- or C4-oxidation as catalytic mechanism.

Genome mining of the LPMOs in fungi showed that the AA9 LPMOs were found mainly in the genomes of asco*-* and basidiomycetes capable of degrading plant biomass whereas genes for AA11 LPMOs were present in the genomes of ascomycetes and in only few of the basidiomycetes.

## Methods

### Genomes

Genome sequences were downloaded from GenBank as indicated (Additional file 3). The classification of the fungi as lignocellulose-degraders was based on description of the fungi as degraders of complex plant cell wall material [33,34]. Hence, brown and white rot basidiomycota and saprophytic ascomycetes able to live on cell walls from dead plants are designated as cellulose-degraders [32-35,37]. On the other hand, *Zygomycota* such as *Thermomucor indicae-seudaticae* were classified as non-degraders based on their classification as fungi growing on easily accessible substrates [59,60].

Overall, the cellulose-degraders included fungi that have evolved to use a broad range of different strategies for biomass decomposition [32,61-63].

### Sequence analysis

Amino acid sequences of all AA9 and AA10 proteins in CAZy [28] were downloaded from GenBank in June 2013. Likewise, AA11 protein sequences [23] were downloaded in January, 2014 and pooled with the GH61 subfamily 7 [24], which includes the same type of proteins as AA11.

Protein domains were mapped in the sequences by CDD search [43] and domains clearly not related to the LPMO enzymatic function, and not overlapping with a relevant domain, were deleted from the sequences. Relevant domains included all domains designated as “Chitin_bind_3”, "Chitin_bind_3 superfamily", "ChtBD1 superfamily", "ChtBD1_1", "Cu_bind_like", "Cu_bind_like superfamily", "Glyco_hydro_61" or "Glyco_hydro_61 superfamily". For proteins belonging to the AA10exp sequence pool domains designated as "COG3397", "COG3979", "Cellulase", "Cellulase superfamily", "ChiA1_BD", "ChiC_BD", "ChtBD3 superfamily", "Collagen", "FN3", "FN3 superfamily", "GH18_chitinase", "GH18_chitinase-like superfamily", "Glyco_18", "PHA03325 superfamily", "PKD", "PKD superfamily" or "PRK13211" were also kept. All sequences that were shorter than 51 amino acids after deletion of unrelated domains were removed.

The curated protein families were analyzed by PPR as previously described for the GH13 and GH61 protein families [24]. The length of the conserved peptides (hexamers), the number of conserved peptides per protein (10) and the total number of conserved peptides per group (70) were chosen as they were the conditions that gave the best rate of prediction of protein function in empirical testing of peptide lengths from trimers to decamers, 5 – 40 conserved peptides per protein and 30 – 200 conserved peptides per group [24].

To generate expanded protein families the top hit in each PPR-generated subfamily of AA9, AA10 and AA11 was used for BLAST search [42] in GenBank. For each of the three AA families, the 1000 best hits of each search were pooled and duplicates were removed. Next, protein domains were mapped in the sequences by CDD search [43] and domains clearly not related to the LPMO enzymatic function were deleted. All sequences shorter than 51 amino acids after deletion of unrelated domains were removed. Furthermore, all AA9-like proteins that were classified as AA11 by PPR search were removed from the AA9 expanded family. Likewise, all AA11-like proteins that were classified as AA9 by PPR search were removed from the AA11 expanded family where after the expanded protein families for AA9, AA10 and AA11 (Additional file 6) were analyzed by PPR as described above.

Finally, the proteins in each subfamily were analyzed by CDD search and manually curated to remove subfamilies that did not include LPMO-like sequences.

### Gene annotation in fungal genomes by finding homology to peptide patterns (Hotpep)

Hotpep analysis was performed as previously described [25]. The procedure can be described by the following steps:

Fungal genomes were split into fragments of 2000 bases with 100 bases overlap. The fragments were translated in all six reading frames and each reading frame was given a score for each subfamily-specific peptide lists for each AA family by:

1. Finding all the conserved peptides from the list that were present in the reading frame.
2. Sum the frequency of these peptides to obtain the subfamily-specific frequency score.

As previously described [25] a hit was considered significant if one of the open reading frames in the sequence:

1. Included three or more conserved peptides from a subfamily.
2. The frequency score for the peptides was higher than 1.0
3. The conserved peptides represented at least ten amino acids of the sequence. (Three hexapeptides with maximal overlap represent 8 amino acids of a sequence whereas three non-overlapping hexapeptides represent 18 (no overlap) amino acids of a sequence).

If a sequence fragment satisfied all three conditions it was assigned to the AA family and to the PPR subfamily with the highest subfamily-specific frequency score as previously described [24]. If two fragments were assigned to the same AA family and the distance between them in the original genome sequence was less than 5800 bases, the fragments were considered to be part of the same gene and counted as one hit.

### Distribution of hexapeptides in the proteins

The position of a conserved hexapeptide was defined as the median of the position in all the protein sequences that contained the hexapeptide sequence. Using the median instead of the average position compensates for the different length of the LPMO sequences and for the occurrence of truncated proteins.

Conserved peptides on the 3D structures were visualized with Cn3D [64].

### Gene annotation in bacterial genomes by finding homology to peptide patterns (Hotpep)

Bacterial genomes were translated in all six reading frames and all open reading frames longer than 50 amino acids were given a score for each subfamily-specific peptide lists for each AA family and significant hits were found as described for gene annotation in fungal genomes.

Each hit was considered as independent of neighboring hits.

### Genome and sequence comparisons

R package version 3.0.1 was used for Ward hierarchical clustering. BLAST search [42] for sequence alignment was done by standard methods and conserved domains were identified in the CDD database at NCBI [43].

### Statistical analysis

Student’s *T*-test was used for comparisons of data sets as indicated.

#### Availability of supporting data

The data sets supporting the results of this article are included within the article and its additional files.

## Additional files

Additional file 1:
**Subfamily distribution of proteins and peptides.** List of GenBank accession numbers for the LPMO proteins in each subfamily and sequence and frequency of the conserved peptides for the subfamily. Additional file 2:
**Cross comparison of peptides in the subfamilies of the expanded LPMO families.** The number of conserved peptides shared between each pair of subfamilies for each of the families AA9exp, AA10exp and AA11exp. Additional file 3:
**Number of LPMO-like sequences found in each fungal genome.** All LPMOs (CAZy auxiliary activity families AA9, AA10 and AA11) were annotated in the genome sequences of the fungi as described in “Methods”. Additional file 4:
**Subfamily distribution of fungal AA9exp, AA10exp and AA11exp.** Number of annotated LPMOs belonging to each AA9exp subfamily, AA10exp subfamily or AA11exp subfamily for each fungal genome. Additional file 5:
**Number of LPMO-like sequences found in each bacterial genome.** All LPMOs (CAZy auxiliary activity families AA9, AA10 and AA11) were annotated in the genome sequences of the bacteria as described in “Methods”. Additional file 6:
**GenBank accession numbers of protein sequences included in AA9exp, AA10exp and AA11exp.** Accession numbers of the protein sequences include the PPR analysis of AA9exp, AA10exp and AA11exp.

## References

[1] Agger JW, Isaksen T, Várnai A, Vidal-Melgosa S, Willats WGT, Ludwig R (2014). Discovery of LPMO activity on hemicelluloses shows the importance of oxidative processes in plant cell wall degradation. Proc Natl Acad Sci U S A.

[2] Forsberg Z, Vaaje-Kolstad G, Westereng B, Bunæs AC, Stenstrøm Y, MacKenzie A (2011). Cleavage of cellulose by a CBM33 protein. Protein Sci.

[3] Harris PV, Welner D, McFarland KC, Re E, Navarro Poulsen J-C, Brown K (2010). Stimulation of lignocellulosic biomass hydrolysis by proteins of glycoside hydrolase family 61: structure and function of a large, enigmatic family. Biochemistry.

[4] Langston JA, Shaghasi T, Abbate E, Xu F, Vlasenko E, Sweeney MD (2011). Oxidoreductive cellulose depolymerization by the enzymes cellobiose dehydrogenase and glycoside hydrolase 61. Appl Environ Microbiol.

[5] Phillips CM, Beeson WT, Cate JH, Marletta MA (2011). Cellobiose dehydrogenase and a copper-dependent polysaccharide monooxygenase potentiate cellulose degradation by *Neurospora crassa*. ACS Chem Biol.

[6] Vaaje-Kolstad G, Westereng B, Horn SJ, Liu Z, Zhai H, Sørlie M (2010). An oxidative enzyme boosting the enzymatic conversion of recalcitrant polysaccharides. Science.

[7] Beeson WT, Phillips CM, Cate JH, Marletta MA (2011). Oxidative cleavage of cellulose by fungal copper-dependent polysaccharide monooxygenases. J Am Chem Soc.

[8] Quinlan RJ, Sweeney MD, Lo Leggio L, Otten H, Poulsen J-CN, Johansen KS (2011). Insights into the oxidative degradation of cellulose by a copper metalloenzyme that exploits biomass components. Proc Natl Acad Sci U S A.

[9] Westereng B, Ishida T, Vaaje-Kolstad G, Wu M, Eijsink VGH, Igarashi K (2011). The putative endoglucanase PcGH61D from *Phanerochaete chrysosporium* is a metal-dependent oxidative enzyme that cleaves cellulose. PLoS One.

[10] Isaksen T, Westereng B, Aachmann FL, Agger JW, Kracher D, Kittl R (2014). A C4-oxidizing lytic polysaccharide monooxygenase cleaving both cellulose and cello-oligosaccharides. J Biol Chem.

[11] Aachmann FL, Sørlie M, Skjåk-Bræk G, Eijsink VGH, Vaaje-Kolstad G (2012). NMR structure of a lytic polysaccharide monooxygenase provides insight into copper binding, protein dynamics, and substrate interactions. Proc Natl Acad Sci U S A.

[12] Li X, Beeson WT, Phillips CM, Marletta MA, Cate JHD (2012). Structural basis for substrate targeting and catalysis by fungal polysaccharide monooxygenases. Structure.

[13] Wu M, Beckham GT, Larsson AM, Ishida T, Kim S, Payne CM (2013). Crystal structure and computational characterization of the lytic polysaccharide monooxygenase GH61D from the basidiomycota fungus *Phanerochaete chrysosporium*. J Biol Chem.

[14] Levasseur A, Drula E, Lombard V, Coutinho PM, Henrissat B (2013). Expansion of the enzymatic repertoire of the CAZy database to integrate auxiliary redox enzymes. Biotechnol Biofuels.

[15] Karlsson J, Saloheimo M, Siika-Aho M, Tenkanen M, Penttilä M, Tjerneld F (2001). Homologous expression and characterization of Cel61A (EG IV) of *Trichoderma reesei*. Eur J Biochem.

[16] Horn SJ, Vaaje-Kolstad G, Westereng B, Eijsink VG (2012). Novel enzymes for the degradation of cellulose. Biotechnol Biofuels.

[17] Leggio LL, Welner D, De Maria L (2012). A structural overview of GH61 proteins - fungal cellulose degrading polysaccharide monooxygenases. Comput Struct Biotechnol J.

[18] Hori C, Igarashi K, Katayama A, Samejima M (2011). Effects of xylan and starch on secretome of the basidiomycete *Phanerochaete chrysosporium* grown on cellulose. FEMS Microbiol Lett.

[19] Ray A, Saykhedkar S, Ayoubi-Canaan P, Hartson SD, Prade R, Mort AJ (2012). *Phanerochaete chrysosporium* produces a diverse array of extracellular enzymes when grown on sorghum. Appl Microbiol Biotechnol.

[20] Yakovlev I, Vaaje-Kolstad G, Hietala AM, Stefańczyk E, Solheim H, Fossdal CG (2012). Substrate-specific transcription of the enigmatic GH61 family of the pathogenic white-rot fungus *Heterobasidion irregulare* during growth on lignocellulose. Appl Microbiol Biotechnol.

[21] Forsberg Z, Røhr AK, Mekasha S, Andersson KK, Eijsink VGH, Vaaje-Kolstad G (2014). Comparative study of two chitin-active and two cellulose-active AA10-type lytic polysaccharide monooxygenases. Biochemistry.

[22] Forsberg Z, Mackenzie AK, Sørlie M, Røhr AK, Helland R, Arvai AS (2014). Structural and functional characterization of a conserved pair of bacterial cellulose-oxidizing lytic polysaccharide monooxygenases. Proc Natl Acad Sci U S A.

[23] Hemsworth GR, Henrissat B, Davies GJ, Walton PH (2014). Discovery and characterization of a new family of lytic polysaccharide monooxygenases. Nat Chem Biol.

[24] Busk PK, Lange L (2013). Function-based classification of carbohydrate-active enzymes by recognition of short, conserved peptide motifs. Appl Environ Microbiol.

[25] Busk PK, Lange M, Pilgaard B, Lange L (2014). Several genes encoding enzymes with the same activity are necessary for aerobic fungal degradation of cellulose in nature. PLoS One.

[26] Stam MR, Danchin EGJ, Rancurel C, Coutinho PM, Henrissat B (2006). Dividing the large glycoside hydrolase family 13 into subfamilies: towards improved functional annotations of alpha-amylase-related proteins. Protein Eng Des Sel.

[27] Aspeborg H, Coutinho PM, Wang Y, Brumer H, Henrissat B (2012). Evolution, substrate specificity and subfamily classification of glycoside hydrolase family 5 (GH5). BMC Evol Biol.

[28] Cantarel BL, Coutinho PM, Rancurel C, Bernard T, Lombard V, Henrissat B (2009). The Carbohydrate-Active EnZymes database (CAZy): an expert resource for Glycogenomics. Nucleic Acids Res.

[29] Lombard V, Golaconda Ramulu H, Drula E, Coutinho PM, Henrissat B (2014). The carbohydrate-active enzymes database (CAZy) in 2013. Nucleic Acids Res.

[30] Busk PK, Lange L. A Novel Method of Providing a Library of N-Mers or Biopolymers. 2012. [WO2012101151].

[31] Hemsworth GR, Taylor EJ, Kim RQ, Gregory RC, Lewis SJ, Turkenburg JP (2013). The copper active site of CBM33 polysaccharide oxygenases. J Am Chem Soc.

[32] Floudas D, Binder M, Riley R, Barry K, Blanchette RA, Henrissat B (2012). The Paleozoic origin of enzymatic lignin decomposition reconstructed from 31 fungal genomes. Science.

[33] Kuhad RC, Kuhar S, Kapoor M, Sharma KK, Singh A (2007). Lignocellulolytic microorganisms, their enzymes and possible biotechnologies based on lignocellulolytic microorganisms and their enzymes.

[34] Moore-Landecker E. Fungi as saprophytes. In Fundamentals of the fungi. Prentice-Hall; New Jersey, USA. 1972. p. 291–325.

[35] Riley R, Salamov AA, Brown DW, Nagy LG, Floudas D, Held BW, et al. Extensive sampling of basidiomycete genomes demonstrates inadequacy of the white-rot/brown-rot paradigm for wood decay fungi. Proc Natl Acad Sci USA. 2014;111:9923-9928. 10.1073/pnas.1400592111 PMC4103376 24958869

[36] Ainsworth GC, Bisby GR, Hawksworth DL (1995). *Ainsworth & Bisby*’*s dictionary of the fungi*. 8^th^ revised edition edition.

[37] Glass NL, Schmoll M, Cate JHD, Coradetti S (2013). Plant cell wall deconstruction by ascomycete fungi. Annu Rev Microbiol.

[38] Baldo A, Monod M, Mathy A, Cambier L, Bagut ET, Defaweux V (2012). Mechanisms of skin adherence and invasion by dermatophytes. Mycoses.

[39] Ng KP, Ngeow YF, Yew SM, Hassan H, Soo-Hoo TS, Na SL (2012). Draft genome sequence of *Daldinia eschscholzii* isolated from blood culture. Eukaryot Cell.

[40] Gianoulis TA, Griffin MA, Spakowicz DJ, Dunican BF, Alpha CJ, Sboner A (2012). Genomic analysis of the hydrocarbon-producing, cellulolytic, endophytic fungus *Ascocoryne sarcoides*. PLoS Genet.

[41] Ohm RA, de Jong JF, Lugones LG, Aerts A, Kothe E, Stajich JE (2010). Genome sequence of the model mushroom *Schizophyllum commune*. Nat Biotechnol.

[42] Altschul SF, Madden TL, Schäffer AA, Zhang J, Zhang Z, Miller W (1997). Gapped BLAST and PSI-BLAST: a new generation of protein database search programs. Nucleic Acids Res.

[43] Marchler-Bauer A, Lu S, Anderson JB, Chitsaz F, Derbyshire MK, DeWeese-Scott C (2011). CDD: a conserved domain database for the functional annotation of proteins. Nucleic Acids Res.

[44] Bey M, Zhou S, Poidevin L, Henrissat B, Coutinho PM, Berrin J-G (2013). Cello-oligosaccharide oxidation reveals differences between Two lytic polysaccharide monooxygenases (family GH61) from *Podospora anserina*. Appl Environ Microbiol.

[45] Vu VV, Beeson WT, Phillips CM, Cate JHD, Marletta MA (2014). Determinants of regioselective hydroxylation in the fungal polysaccharide monooxygenases. J Am Chem Soc.

[46] Vaaje-Kolstad G, Bøhle LA, Gåseidnes S, Dalhus B, Bjørås M, Mathiesen G (2012). Characterization of the chitinolytic machinery of *Enterococcus faecalis* V583 and high-resolution structure of its oxidative CBM33 enzyme. J Mol Biol.

[47] Gudmundsson M, Kim S, Wu M, Ishida T, Momeni MH, Vaaje-Kolstad G (2014). Structural and electronic snapshots during the transition from a Cu(II) to Cu(I) metal center of a lytic polysaccharide monooxygenase by X-ray photoreduction. J Biol Chem.

[48] Monod M (2008). Secreted proteases from dermatophytes. Mycopathologia.

[49] Martinez DA, Oliver BG, Gräser Y, Goldberg JM, Li W, Martinez-Rossi NM (2012). Comparative genome analysis of *Trichophyton rubrum* and related dermatophytes reveals candidate genes involved in infection. MBio.

[50] Zaugg C, Monod M, Weber J, Harshman K, Pradervand S, Thomas J (2009). Gene expression profiling in the human pathogenic dermatophyte *Trichophyton rubrum* during growth on proteins. Eukaryot Cell.

[51] Loftus BJ, Fung E, Roncaglia P, Rowley D, Amedeo P, Bruno D (2005). The genome of the basidiomycetous yeast and human pathogen *Cryptococcus neoformans*. Science.

[52] Baron O, Cassaing S, Percodani J, Berry A, Linas M-D, Fabre R (2006). Nucleotide sequencing for diagnosis of sinusal infection by *Schizophyllum commune*, an uncommon pathogenic fungus. J Clin Microbiol.

[53] Tanaka H, Takizawa K, Baba O, Maeda T, Fukushima K, Shinya K (2008). Basidiomycosis: *Schizophyllum commune* osteomyelitis in a dog. J Vet Med Sci.

[54] Zugmaier W, Bauer R, Oberwinkler F (1994). Mycoparasitism of some tremella species. Mycologia.

[55] Fitzpatrick DA (2012). Horizontal gene transfer in fungi. FEMS Microbiol Lett.

[56] Jumpponen A, Jones KL (2010). Seasonally dynamic fungal communities in the *Quercus macrocarpa* phyllosphere differ between urban and nonurban environments. New Phytol.

[57] Redford AJ, Bowers RM, Knight R, Linhart Y, Fierer N (2010). The ecology of the phyllosphere: geographic and phylogenetic variability in the distribution of bacteria on tree leaves. Environ Microbiol.

[58] Spring J (2003). Major transitions in evolution by genome fusions: from prokaryotes to eukaryotes, metazoans, bilaterians and vertebrates. J Struct Funct Genomics.

[59] Richardson M (2009). The ecology of the Zygomycetes and its impact on environmental exposure. Clin Microbiol Infect.

[60] Busk PK, Lange L (2013). Cellulolytic potential of thermophilic species from four fungal orders. AMB Express.

[61] Busk PK (2013). The meaning of life - On cactus finches, evolution and chaos.

[62] Van den Brink J, de Vries RP (2011). Fungal enzyme sets for plant polysaccharide degradation. Appl Microbiol Biotechnol.

[63] Sánchez C (2009). Lignocellulosic residues: biodegradation and bioconversion by fungi. Biotechnol Adv.

[64] Wang Y, Geer LY, Chappey C, Kans JA, Bryant SH (2000). Cn3D: sequence and structure views for Entrez. Trends Biochem Sci.

