# Supplementary material for: Classification of fungal and bacterial lytic polysaccharide monooxygenases
Source: BMC Genomics. 2015 May 9;16(1):368. doi: 10.1186/s12864-015-1601-6 (PMC4424831; doi:10.1186/s12864-015-1601-6)
Supplement: Additional file 6: — GenBank accession numbers of protein sequences included in AA9exp, AA10exp and AA11exp. Accession numbers of the protein sequences include the PPR analysis of AA9exp, AA10exp and AA11exp. [file 12864_2015_1601_MOESM6_ESM.docx]

**Additional file 6.txt: GenBank accession numbers of protein sequences included in AA9exp, AA10exp and AA11exp.**

**GenBank accession numbers of protein sequences included in AA9exp.**

XP_003112607.1, NP_001245494.1, NP_001245491.1, NP_001245493.1, NP_001162648.2, NP_001027038.2, NP_001245500.1, EPE05023.1, AER12034.1, NP_001265728.1, EGX48824.1, EPS44425.1, WP_003699136.1, EPS35788.1, XP_003307059.1, XP_002835340.1, EMC94952.1, EKD20061.1, EHK27308.1, XP_005302725.1, XP_005302726.1, XP_661115.1, EPE27905.1, EPS36332.1, ELU42320.1, EQC36258.1, EPS44617.1, ENH80456.1, XP_003288165.1, EKJ75367.1, XP_005767260.1, XP_388088.1, XP_006691631.1, ELU39649.1, ELQ42903.1, WP_003780339.1, ETT55145.1, EPE28396.1, EGG03285.1, XP_001228820.1, YP_616331.1, EFY98227.1, XP_004555290.1, XP_004555291.1, ELU42878.1, XP_005476743.1, XP_005476744.1, XP_005729072.1, XP_005729073.1, ERF74842.1, XP_003837668.1, XP_004555292.1, XP_005924623.1, XP_005729075.1, XP_003026963.1, ELA32570.1, XP_005729074.1, ACE10235.1, XP_003711925.1, YP_003950080.1, XP_005729076.1, YP_004902260.1, WP_002618044.1, YP_006758328.1, ERF68713.1, XP_001227970.1, XP_003298498.1, XP_659992.1, EPS35677.1, ENH80953.1, WP_009613634.1, XP_003835032.1, EGX51594.1, XP_001930436.1, EKD00284.1, EKD19627.1, EJT45344.1, EUC50628.1, EGO52648.1, EUC35350.1, EUN30555.1, EMD69380.1, EPQ57572.1, XP_001941054.1, XP_964574.1, EGZ77475.1, EMD95747.1, EUC57419.1, XP_001799040.1, EON98488.1, ACE10233.1, GAA99777.1, EGU84223.1, XP_003302923.1, ENH80646.1, XP_006693659.1, XP_003348785.1, XP_003348396.1, CCF33561.1, EUC44375.1, XP_001802011.1, EMD60643.1, EUC61453.1, XP_003837405.1, XP_755782.1, EFQ30762.1, EUC31954.1, EKJ77293.1, XP_386384.1, EKD20231.1, EIM80235.1, EMT60607.1, ENH69113.1, EOA90071.1, EJT78526.1, XP_001834768.1, ELU36500.1, XP_003045940.1, CCT63889.1, XP_003299166.1, EUC45358.1, EUC26826.1, EUN21440.1, XP_005775219.1, XP_001939155.1, ACE10232.1, EON60916.1, EKG12135.1, EOA86940.1, EOD51280.1, XP_962644.1, XP_001260907.1, EGX54086.1, XP_005776160.1, EGO59472.1, XP_001909116.1, XP_006693581.1, EMR61459.1, CCA72221.1, EUN24691.1, CCF38904.1, EJD37625.1, ACE10234.1, XP_006672376.1, ESK88398.1, EFQ35966.1, EFQ34935.1, ETI21170.1, EFX04819.1, EME48042.1, XP_003661021.1, EMD90199.1, EIM83161.1, EGG08525.1, XP_001590753.1, CCD52645.1, XP_003037985.1, CAK38942.1, GAA90961.1, EGY19267.1, XP_658645.1, ESZ97680.1, EQB58806.1, ENI09589.1, YP_002462169.1, ELR08548.1, EKD21599.1, EPS40687.1, WP_022120710.1, WP_006846640.1, ELU37145.1, BAE58643.1, CAK41095.1, EGX50880.1, EKD12576.1, ETS83207.1, EIM23466.1, YP_001378623.1, ENH87373.1, CBF87153.1, XP_003006393.1, XP_001275694.1, EIM91666.1, EKD16460.1, EOA87425.1, EPS38835.1, EPE33109.1, EGY13846.1, EHY59340.1, ENH81324.1, EQB45742.1, ENH81697.1, EIM91642.1, A2R5N0.1, GAA87040.1, EUC55193.1, EGG03321.1, Q96WQ9.1, EJD00880.1, XP_003028465.1, GAD94768.1, EUC56027.1, EJP65452.1, EOR04231.1, XP_003890761.1, EPS43636.1, EGG05780.1, WP_022327106.1, XP_001392640.1, XP_868906.1, EPE24990.1, XP_001210534.1, ETS87426.1, NP_001130665.1, GAA88487.1, EJT77153.1, EUC55821.1, EGU12834.1, EMS18616.1, EIM79875.1, XP_002381515.1, ERT01561.1, XP_001825528.1, XP_003026867.1, EOQ99960.1, XP_003031883.1, CCO33227.1, XP_001402213.2, EPE31355.1, CCX08366.1, EQB56202.1, CAP92380.1, ELA29460.1, XP_001208400.1, CCX10545.1, ETS87915.1, XP_003855239.1, EHK97353.1, EUC58402.1, EME85870.1, ENI00713.1, CCU74678.1, EPQ65317.1, EKG10268.1, EHL01403.1, XP_003032377.1, ETN42574.1, XP_001834810.1, XP_003336129.1, ADX07320.1, ETS73214.1, EPE09983.1, EPS25762.1, XP_003834319.1, XP_002840464.1, YP_006250136.1, EKM51119.1, ETN42051.1, XP_001400893.2, XP_001267517.1, EPT02242.1, WP_022285453.1, ELQ34553.1, EOD49346.1, CCE28482.1, EJD55491.1, EAT77212.1, EMC96205.1, EOD48576.1, ETS03449.1, CCX15015.1, EPS43405.1, EMR69551.1, EGY16057.1, XP_003325408.1, ELU38904.1, XP_001553245.1, ESK93339.1, XP_003044062.1, AFR92731.2, XP_003710208.1, XP_002999756.1, ESK93407.1, EUC56891.1, EUC60599.1, XP_752626.1, EMR66261.1, XP_003836911.1, ENH65124.1, CCT67584.1, EHK43620.1, EMT66946.1, XP_002380189.1, EFY92676.1, XP_001820645.2, XP_001216541.1, XP_003349158.1, CCX14364.1, XP_003043569.1, XP_003838016.1, EGU75413.1, XP_001903701.1, EGX44929.1, XP_003191597.1, EHK23181.1, EKG11261.1, XP_001818766.1, XP_958254.1, EOR02787.1, XP_003650513.1, XP_003717521.1, ESU14240.1, XP_003653732.1, XP_001264507.1, XP_002372701.1, XP_006457450.1, EKM77532.1, XP_681160.1, EPS95842.1, AFR92731.1, EPS35120.1, XP_566940.1, XP_001397023.2, EMD64966.1, EGZ76369.1, CCO27431.1, XP_777868.1, EMD92020.1, XP_001584877.1, XP_001790904.1, EDP47167.1, GAD95029.1, ETS75420.1, EGX45639.1, CBF73467.1, EJD52178.1, EGX53143.1, EMT66504.1, EGU80176.1, EMR71424.1, EKV06084.1, XP_001799980.1, CCF40964.1, CCA74814.1, EFX04021.1, XP_001936366.1, XP_001216411.1, EIW75818.1, AGT80113.1, XP_384949.1, XP_003834678.1, EGO53330.1, EJT73001.1, EAA36362.1, CCX04393.1, ETS73538.1, EPS45974.1, XP_001817584.2, ENH67075.1, ENH74816.1, NP_001141352.1, CCT72465.1, EGU87361.1, XP_003042567.1, XP_001936606.1, XP_659206.1, ENH65812.1, EKM51828.1, EGU74313.1, CCT73805.1, EMT65716.1, EUC35230.1, EOR01204.1, EPS36434.1, EOR04232.1, EHA24746.1, ESZ91697.1, EUC43657.1, XP_001836535.2, EPS26325.1, EMR68134.1, XP_001273555.1, EGO61608.1, EFQ34588.1, XP_384144.1, EGY23592.1, EHA18110.1, XP_001276738.1, XP_001834690.1, , EGX53531.1, ELU40254.1, XP_003000578.1, XP_003027612.1, XP_003301611.1, EIN11131.1, XP_001911429.1, XP_002558168.1, EFQ36324.1, XP_001213388.1, EMD41283.1, , XP_001791868.1, EJF60634.1, XP_006694464.1, EMD68819.1, XP_001931381.1, EGU84205.1, CAK46515.1, XP_001225249.1, EUC55046.1, EHK46784.1, EPS37353.1, XP_747482.1, B0Y9G4.1, EMR72626.1, ENH65939.1, CCL99937.1, EKD14766.1, XP_006457452.1, XP_001259147.1, XP_001262213.1, EIM92983.1, XP_003346899.1, ETN43948.1, , ACH92573.1, AFD50197.1, XP_003303895.1, XP_003349072.1, EHK19374.1, EOD45519.1, EUN25397.1, ENH88713.1, EQB51578.1, ELA32496.1, XP_001214259.1, XP_001591031.1, EIM80104.1, XP_006692963.1, EON99761.1, EPS44225.1, GAA90386.1, ADB89217.1, EKM57567.1, ENH85732.1, EQB52166.1, XP_001906078.1, XP_001912223.1, EIN08692.1, EKG19150.1, XP_001225692.1, EGO53245.1, CAA71999.1, EMD35593.1, EAA36262.1, ADJ57703.1, EUN27858.1, EMT72483.1, EPQ61333.1, EKM77534.1, EOA87924.1, ESW27017.1, EMD95968.1, EKM60167.1, EUC50443.1, EMD69294.1, EUC35884.1, XP_001269010.1, EUN30631.1, EPE03845.1, EMD84760.1, EGZ78102.1, EJD42501.1, XP_003661787.1, EGO58503.1, XP_748707.1, EKD20293.1, XP_956109.1, ETW79866.1, CCT72729.1, EGU72891.1, XP_001832109.2, XP_002474815.1, AFO72237.1, XP_001832511.1, EED85774.1, XP_001934008.1, ETT88246.1, NP_830205.1, WP_000756603.1, EUC45146.1, WP_000756600.1, EIW63682.1, ENH76919.1, EJF58529.1, EJT69796.1, EHA27737.1, EGO54705.1, EJC99207.1, EIN14614.1, XP_958583.1, EIN03477.1, EKJ74557.1, EGX50455.1, EIN08693.1, EIW75816.1, CCT64954.1, EUC31685.1, EKJ79308.1, XP_001396992.1, EGX45391.1, XP_003664166.1, EMD64084.1, ETN45589.1, EGU86650.1, CCT64153.1, XP_006692663.1, EOA89857.1, ENH65661.1, EJT68517.1, EIW65370.1, ELU45307.1, EMT60290.1, EJF62088.1, ENH83985.1, EUC66210.1, XP_001932895.1, XP_003663657.1, XP_001840891.1, EJT71428.1, EPE07594.1, EGY21435.1, EKJ77976.1, EKJ72039.1, EJD03131.1, XP_003711808.1, EJT69484.1, XP_001933725.1, EJF65292.1, EMD60067.1, XP_003711556.1, EPS41590.1, XP_001258070.1, XP_382378.1, EJF62118.1, EGX47430.1, EMD97722.1, XP_003653731.1, EUN24558.1, EJC97864.1, EUC26970.1, CCF37197.1, EOA81141.1, XP_001832512.1, EUC46849.1, XP_001934763.1, ENH86172.1, XP_003716689.1, XP_003296033.1, ETS76507.1, EON96051.1, XP_003034283.1, CCA67659.1, EMD35749.1, AFO72239.1, ETW78476.1, EJT76558.1, XP_001883194.1, EQB51904.1, ETN46770.1, XP_002626386.1, EPS43036.1, EPS43624.1, EEQ84968.1, ERS99039.1, EGE79257.1, AFJ54163.1, ABT35335.1, EQL35734.1, EIW52742.1, EIM24026.1, EGX47847.1, CCA72220.1, XP_003345284.1, EAA26873.2, EQB58826.1, ETS75650.1, XP_750843.1, XP_003655099.1, EOA90805.1, AAM22493.1, EOA86603.1, EJD51746.1, XP_001873748.1, EIM23310.1, CCO35238.1, EFQ25762.1, EUC60645.1, EOO02157.1, EOA80928.1, EJT71331.1, XP_001804665.1, CCO32147.1, XP_001826694.1, EGX52397.1, EGU79199.1, EUN24871.1, CAP68375.1, ERS95549.1, EUC45736.1, EUC35438.1, EMT71116.1, EGX53328.1, EUC66211.1, XP_003655380.1, XP_001229931.1, EPS38958.1, EPS40743.1, XP_963702.1, ELQ44326.1, EMD61937.1, WP_007819893.1, XP_003299575.1, ELA33045.1, EMR87298.1, EGX48532.1, XP_001553240.1, EPE31098.1, XP_003841733.1, EJC98512.1, EOA84098.1, XP_005788994.1, ENH88859.1, XP_001797837.1, XP_006460845.1, CCO29552.1, EPS44424.1, EPS42243.1, EKM80723.1, CCA67657.1, XP_003663414.1, EQB52147.1, EQB45245.1, EMT66616.1, EUC44040.1, XP_003351373.1, XP_001906521.1, EMD60707.1, XP_003302575.1, XP_959499.1, ENH78567.1, EUC34066.1, EUN24218.1, XP_001933274.1, EJT69787.1, ESZ91699.1, XP_001835755.1, EGX48168.1, XP_006692680.1, CCA72192.1, EGO19770.1, EMR82768.1, CCT74544.1, CCD34368.1, XP_003303817.1, EMR84214.1, XP_001589530.1, EPQ53386.1, ETW75575.1, XP_003046131.1, EJF62185.1, EJD47911.1, AFO72234.1, CCD49290.1, XP_001841073.2, ELA25859.1, XP_003856492.1, EQB59443.1, EUC40396.1, EHL02929.1, EMD31057.1, EMD31056.1, EGX52740.1, Q00023.1, XP_002478590.1, CCO35344.1, EKD11921.1, EFQ25116.1, EJF61089.1, EJD44963.1, EUC57420.1, EIM86349.1, EON66774.1, ENH83348.1, ESZ90446.1, AAM76663.1, CAG27577.1, ETS86148.1, XP_001936296.1, EKJ71808.1, CCD50144.1, XP_001584875.1, EIN12107.1, ETS78148.1, EIW59705.1, XP_391664.1, EJT78003.1, EGY14544.1, EKM51116.1, XP_003654359.1, EUC36210.1, EMD70171.1, ETS78657.1, EPS45603.1, EPE27438.1, ESK96629.1, EFQ35295.1, EKM56323.1, EGX44104.1, XP_001840081.2, EJD53151.1, XP_001907679.1, EIW61516.1, EJD47200.1, EIW77477.1, EMD86456.1, EPS31810.1, EUN32562.1, EJD08026.1, CAQ16208.1, ESK88334.1, EFQ28963.1, XP_369715.2, XP_003719782.1, AFO72238.1, ETW81695.1, XP_001587030.1, ETS86000.1, EIW59704.1, XP_006690939.1, EMR66616.1, EFQ31130.1, CCA71764.1, XP_002146302.1, ELU45255.1, EGN94280.1, EJD34799.1, ELA29827.1, CCO33433.1, EMR70379.1, CCF42122.1, XP_001727159.1, CCL98501.1, XP_002474489.1, EKJ67485.1, ETS73783.1, ERF74952.1, CCO34933.1, XP_003000367.1, EFQ36423.1, EUC55826.1, CCA70703.1, XP_001222797.1, CCF41607.1, AAT64005.1, XP_001589188.1, EGX51003.1, ENH83249.1, XP_006690646.1, EKM75124.1, EMF15548.1, ESK95764.1, XP_958368.1, EGO58321.1, EQB58232.1, XP_001841257.2, XP_001832515.2, XP_386573.1, CCA67656.1, XP_003345539.1, EUC58649.1, EJT79766.1, XP_367664.2, EFQ27394.1, CCA72183.1, , XP_001225412.1, AEO56416.1, CCA75037.1, EKM81411.1, CCO37025.1, XP_001560175.1, CCD47228.1, EFQ34537.1, EOD52980.1, EJT74901.1, EUC57405.1, EOA87074.1, XP_003001513.1, CCA72182.1, ELQ40404.1, XP_001274885.1, EQB58273.1, XP_003659754.1, ENH77138.1, EUC54563.1, GAD92924.1, ETN44819.1, AGT80098.1, EFQ36570.1, XP_001558279.1, EGY17063.1, EJD47947.1, XP_001834453.2, XP_003352682.1, AEX63130.1, XP_001904712.1, EUC28180.1, EUN21797.1, XP_003007169.1, EGN94279.1, EMD69877.1, YP_007354141.1, EIM85952.1, CCX05799.1, XP_001804100.1, EMR72638.1, XP_001840824.2, XP_001219301.1, XP_001903620.1, ELA38153.1, EHL01858.1, XP_003000747.1, CCF46377.1, ENH67995.1, EMT69567.1, CCT75380.1, XP_001209496.1, EGY16236.1, EUC39752.1, XP_001226408.1, XP_001802947.1, XP_001227307.1, EIM20521.1, CCF39722.1, XP_001404656.1, XP_388187.1, EGY20195.1, XP_003335269.1, XP_001222154.1, XP_001883867.1, XP_001223598.1, EOR00085.1, XP_003715649.1, XP_001225332.1, EGO52064.1, XP_001939406.1, CAQ16217.1, ENH86303.1, EGY23357.1, XP_959466.1, XP_001907069.1, ELQ39199.1, EMR67698.1, XP_001226997.1, XP_001933351.1, EOA83841.1, EPE27481.1, EMR87655.1, EKJ77489.1, XP_361583.1, EGZ76872.1, EMR61887.1, EKM58977.1, ESZ89490.1, XP_001260913.1, EOA89438.1, XP_003844683.1, XP_001931371.1, EGU11067.1, EKG19237.1, XP_755787.1, EMD67812.1, CCA70418.1, EFQ33676.1, ETS81017.1, EIM19648.1, EOQ98927.1, XP_003300990.1, EMD92059.1, XP_003009235.1, CCA74449.1, EUC42683.1, XP_660650.1, ETS73115.1, XP_001907502.1, CCC07649.1, EIM21082.1, CCF43462.1, EUC29084.1, XP_001796146.1, EIW52776.1, EMS20069.1, XP_001224238.1, EOR04760.1, XP_003345666.1, EFQ35556.1, EQB48191.1, EMD86148.1, XP_002376462.1, XP_002563599.1, EUC58572.1, XP_001820737.1, XP_386286.1, EUC44341.1, EGY15511.1, ERS95126.1, EJT82373.1, XP_003296793.1, EUN22444.1, XP_001208821.1, EMD64743.1, EMD88231.1, EUC36549.1, XP_001274726.1, XP_001405918.1, XP_001795846.1, EOA82348.1, EME80701.1, XP_001395530.1, EKG16536.1, GAA86473.1, ERS95399.1, XP_001791546.1, XP_001911495.1, EUC58700.1, EGU85487.1, EMT68323.1, XP_003050495.1, EIW78453.1, EGO03895.1, EIW74395.1, EIW54867.1, EKJ78901.1, ESU12158.1, EGY21971.1, ELQ36889.1, CCF38942.1, EOD44350.1, CAJ81217.1, EHL00451.1, ELQ44924.1, XP_003299949.1, XP_003040612.1, XP_001222217.1, EJC98672.1, CBF76006.1, XP_003709110.1, ELU45811.1, EFQ30363.1, XP_003714973.1, EME47180.1, XP_001395335.2, EMR70311.1, ELA28341.1, ERS96554.1, EIM22136.1, CCT67119.1, EJT72133.1, XP_001597642.1, EKD16174.1, XP_001558907.1, XP_001220412.1, ENH81876.1, EKJ77390.1, ELA38278.1, EPE30977.1, XP_001222998.1, XP_001840838.2, XP_003837976.1, EUC45340.1, EJT76773.1, EMD60622.1, EMD90222.1, EOA90016.1, XP_003003091.1, EUC32814.1, ERT00522.1, XP_384857.1, XP_001217528.1, EFQ29051.1, XP_003301020.1, XP_003045670.1, EPE28947.1, EKM52808.1, EME86418.1, XP_001834459.2, XP_001801858.1, XP_001930525.1, ETS83233.1, ELA27328.1, EMT71117.1, EUC47439.1, EGY21381.1, ESZ98571.1, EQB50782.1, CAJ81216.1, XP_003351982.1, EKG21093.1, EGY22966.1, EOD52617.1, CAQ16278.1, EOD52343.1, XP_003005258.1, XP_003026941.1, XP_001802269.1, XP_001834458.1, EMR69581.1, ENH68598.1, EMR61986.1, ELA27522.1, EMR70221.1, XP_001219444.1, XP_006693924.1, XP_001832374.2, EON95758.1, CCO33440.1, EOA88391.1, EMD39566.1, ELA25729.1, XP_003717642.1, ELA35512.1, XP_369714.1, EMT72114.1, EPE04001.1, ETS73547.1, XP_003002008.1, CCT72942.1, ENH75982.1, EGY20283.1, EQB59267.1, ERT00531.1, XP_003655379.1, EHL01835.1, EGY15740.1, CCF31859.1, EIW53153.1, XP_001792546.1, XP_003661910.1, XP_001904958.1, XP_002838651.1, CCO33441.1, EFQ35170.1, CAQ16206.1, EIW62895.1, XP_006454828.1, EGU75302.1, XP_003346672.1, CCA94932.1, XP_001911486.1, EJC98256.1, EMR87398.1, XP_003351237.1, EKD16974.1, EQB50615.1, EMR70622.1, XP_003840029.1, XP_003001445.1, ELA38372.1, EJF65162.1, XP_003665068.1, EPE30726.1, EMF15770.1, CCO32359.1, CCF47000.1, EIM86641.1, CAP71839.1, EKG11468.1, EPE30275.1, EPE24515.1, ENH76818.1, EMR69607.1, ENH78917.1, AFO72235.1, AEJ35168.1, EQB46141.1, EMR69712.1, ESK92658.1, XP_003841197.1, EMR64861.1, ETW78724.1, CCF36552.1, ELA23652.1, EQB47908.1, EPE37061.1, EMD94974.1, ESK83440.1, XP_001596818.1, CCA70417.1, XP_001937216.1, EJD34609.1, EHK19935.1, EHK50961.1, ELA36476.1, XP_001940528.1, EQB49523.1, EFQ36036.1, XP_001224181.1, XP_003654913.1, EOA85303.1, ABW56451.1, ETS76862.1, EMD66246.1, EUN23843.1, XP_752040.1, ESK95156.1, EUC46665.1, XP_001821292.1, XP_001271679.1, XP_001792455.1, EGN93952.1, ELA35778.1, XP_001230208.1, CCD50451.2, XP_001267236.1, XP_661464.1, ELA29632.1, EUC39233.1, XP_003031059.1, EIW81812.1, EAU83857.1, ACD36971.1, ETS73035.1, AGO68294.1, CAP67176.1, Q7Z9M7.3, XP_003720165.1, EUC64196.1, XP_001800354.1, EPE24460.1, EGD97724.1, ESK91695.1, EPS45349.1, EPS45954.1, CCO27159.1, ETS85670.1, EPS30681.1, EUC60768.1, EMR72556.1, XP_003300983.1, EPE08322.1, XP_003715529.1, EIW82923.1, EKM81001.1, ERT03091.1, EIN05067.1, EGX46833.1, XP_001935843.1, ETW84871.1, EGN99889.1, EOA87287.1, XP_006461132.1, EON98508.1, CCF37367.1, EMR63023.1, EMT70765.1, EIW81877.1, XP_003345982.1, EUC32112.1, XP_003045244.1, EIN08952.1, EUN24457.1, EMD65024.1, XP_003033157.1, EMD87940.1, ENH66119.1, ELA24367.1, CCT69268.1, EUC60659.1, EQB50095.1, ETS77995.1, EKJ71197.1, EJD49498.1, EOD44449.1, EUC47338.1, CCA94933.1, XP_001546354.1, ENH83051.1, EUC56717.1, EUC55036.1, XP_001905728.1, EON99293.1, EMR69162.1, XP_003661261.1, CCA73151.1, CAB97283.2, XP_001910915.1, EOA84740.1, EFQ34827.1, ENH82205.1, EQB53887.1, XP_003648758.1, EUC37964.1, ENH76298.1, EMR90953.1, XP_001219904.1, ESK89694.1, CCX07706.1, EHA20242.1, XP_003845453.1, EGS18626.1, ELA36489.1, EIM85306.1, EFQ33926.1, XP_003296866.1, XP_002559170.1, XP_001932318.1, EOR00736.1, EOD48151.1, EJF60005.1, EQB43879.1, EIW81813.1, ESK85191.1, EGY16927.1, XP_003034906.1, EMD66687.1, ELU45298.1, XP_001838602.1, EUC49560.1, EIM21862.1, EMD97281.1, XP_003661792.1, XP_003007405.1, ELA29128.1, XP_003300926.1, ETW87087.1, EPE26434.1, EIN04298.1, EJF65049.1, EJT76774.1, XP_003841605.1, EIW62723.1, AFO72233.1, XP_001936652.1, EJD55170.1, EUC40397.1, EMD68959.1, EMR68578.1, EJD51748.1, XP_383871.1, ESK97120.1, EOA89611.1, CCF33897.1, EKG18949.1, EIM24072.1, CAP61048.1, EGS17857.1, XP_001230192.1, EFQ31502.1, EIW62371.1, XP_001225243.1, EGU77464.1, EKJ77911.1, EIW62722.1, XP_001230041.1, EMD96915.1, EMD68707.1, EGX46140.1, EUC39364.1, EUC48418.1, ESK95765.1, EGU85118.1, XP_003711630.1, CCT74587.1, XP_003660897.1, EGO58845.1, EHL00250.1, XP_002389159.1, EJT70262.1, EIW59703.1, EGY16992.1, EGX44040.1, XP_003029596.1, XP_962414.1, XP_003666502.1, EPE27226.1, XP_003002719.1, EGY21187.1, XP_001905203.1, XP_003652610.1, EGO53412.1, ETW81696.1, EGZ76445.1, EAA36150.2, EON98840.1, AFO72236.1, EOQ99434.1, ETS82901.1, ENH89337.1, EQB54916.1, XP_003839955.1, EMT69565.1, XP_002391345.1, XP_003651916.1, EPE03095.1, CCO29715.1, ERT03352.1, XP_001907637.1, EJD05308.1, EJT77316.1, EJF61090.1, EUC29591.1, XP_001410346.1, EUC47278.1, EMD88003.1, EIM81969.1, XP_001832364.2, EFQ34021.1, XP_001225107.1, EUC29594.1, EMD88000.1, XP_003041666.1, XP_001216734.1, CCA94930.1, XP_003352294.1, XP_003304335.1, EMD64969.1, EGO61029.1, EPE30131.1, EDU48860.1, CAD21296.1, CCA70035.1, EAA33178.2, CCA94931.1, EIN11130.1, EHL03008.1, EUC47281.1, ENH76153.1, EPT02783.1, EIW61432.1, EIM86332.1, XP_003661887.1, EIM89471.1, ELQ35338.1, EJF63152.1, ENH80826.1, EUC57812.1, XP_001905612.1, EKM57537.1, EPQ59852.1, CCA77877.1, EMR66609.1, XP_001223312.1, CCF46308.1, EGS17558.1, CCO26437.1, EGO55727.1, XP_001911247.1, XP_003027507.1, ELR02062.1, EOA89951.1, ESK94064.1, XP_006456875.1, EMR62200.1, ESK92657.1, EUC56860.1, CDJ79823.1, EKM79816.1, ESK95698.1, XP_001227193.1, EGY16544.1, EGX43865.1, EMR66955.1, XP_367775.2, CBY01257.1, XP_003041587.1, XP_003029697.1, BAL43430.1, EMD35431.1, XP_001225324.1, XP_003002463.1, EMD35432.1, XP_003665200.1, CCO32372.1, EMS20008.1, EIW55288.1, EMD35519.1, EIN14513.1, EIN14512.1, EJF57368.1, EPE29707.1, AEQ16462.1, CCO37880.1, EUN27096.1, XP_003349473.1, EUC55601.1, CCO37929.1, EJD43461.1, EGO55619.1, EMD90911.1, EUC33351.1, EAQ70783.1, XP_001906795.1, EJT73807.1, EMD67389.1, ESK95699.1, XP_003716906.1, EFQ27332.1, ELA29255.1, EPS97565.1, EQB53449.1, EGX44454.1, ELA28325.1, EKM57538.1, EMD92474.1, EUC46203.1, CAJ81215.1, XP_002910838.1, ESK86393.1, AEO67662.1, EUC41372.1, EGY15143.1, XP_001801791.1, XP_003305323.1, AFP23133.1, EUC58686.1, XP_001905623.1, CCA94934.1, ETW77353.1, EUC27622.1, EMR61956.1, ENH79117.1, EGS19451.1, EAT81480.1, ETS81471.1, XP_001909024.1, EMD65966.1, EOD42834.1, CCF45462.1, EMR66073.1, ESK86392.1, EOA85849.1, EMD89537.1, EQB46954.1, ETW77401.1, AFO72232.1, EJT75180.1, XP_001224551.1, EJD03601.1, XP_003665516.1, XP_003031141.1, CCO37016.1, EFQ25679.1, XP_001214594.1, EIW53754.1, EOO00823.1, EGO56889.1, XP_003839134.1, EUC61095.1, EJD54109.1, XP_001841005.1, EIM84462.1, EQB48529.1, XP_003297809.1, EPE33840.1, XP_001906822.1, XP_001841004.1, XP_664032.1, XP_001832108.1, EGO19398.1, EFQ34070.1, EOD45458.1, XP_955892.1, EQB47934.1, ELA32320.1, ELA26365.1, CCX08816.1, XP_003026965.1, XP_003666550.1, ERS97831.1, ERT01930.1, XP_001225930.1, XP_001215255.1, XP_001791556.1, XP_001937817.1, EMR72715.1, XP_003657367.1, EPE10402.1, XP_006463428.1, EUC61724.1, CCO27748.1, XP_003840428.1, XP_003305915.1, XP_003027567.1, ERT03386.1, ESK86397.1, EKM77379.1, XP_003346673.1, EMR67821.1, XP_003027576.1, XP_001790826.1, XP_003306650.1, EMD87585.1, CCA73144.1, XP_003037795.1, XP_003709306.1, EUC38446.1, ETS77716.1, XP_001933987.1, EUC48914.1, EJF57191.1, EMD59230.1, EOA88408.1, EPS41576.1, CCD51504.1, CCA67658.1, ETS73220.1, EPS36316.1, EMR84283.1, XP_003004661.1, XP_003715832.1, AEV53599.1, XP_001791800.1, EIW61483.1, XP_001219583.1, 2YET, XP_006693905.1, EGX43029.1, EPS35228.1, EMC92772.1, EGY17523.1, XP_001791488.1, CCF31858.1, EHK96369.1, EFQ34071.1, XP_003654493.1, XP_003665081.1, EGY15133.1, XP_001227732.1, XP_003351260.1, EPE25549.1, EOR00122.1, CCA74246.1, EIM19728.1, XP_001224473.1, XP_003660327.1, ELA28967.1, EJD52914.1, XP_006691284.1, XP_001882504.1, CCO29835.1, XP_001225931.1, CAJ81218.1, ERT01158.1, AEO61304.1, CAP64732.1, EOD42993.1, EMR72190.1, XP_001223687.1, EHL02213.1, 4EIS, EOO01726.1, CAP67481.1, ELA26364.1, 4EIS, EMR69548.1, EKM57539.1, ERS97419.1, EJT72948.1, EJT72853.1, XP_003349115.1, EAA50788.1, ETS75884.1, EGY18914.1, XP_003845721.1, EPE27225.1, 4EIR, EJD45118.1, XP_003650941.1, EOA87389.1, ETS81778.1, CCO32571.1, EJD44215.1, XP_001903874.1, EUC37952.1, EMD97272.1, EMD66678.1, XP_001935377.1, XP_001932393.1, EUC49551.1, XP_003299155.1, XP_001793286.1, XP_003043105.1, XP_001835420.2, EMR69141.1, XP_001408162.1, ESK86498.1, EOA84748.1, ETS85458.1, XP_003715019.1, EUC55313.1, CCX30902.1, EOD51028.1, EOA87752.1, CCO32944.1, XP_001835421.1, EUC55091.1, EUC42150.1, XP_001805107.1, XP_001841512.1, EMD62481.1, EUN21481.1, EUC27573.1, XP_001552214.1, EMD93828.1, CAG27578.1, EIM85185.1, EJD07961.1, XP_001885903.1, XP_006456576.1, ESK86499.1, EKM76292.1, XP_001839611.1, EKM76822.1, EIN11198.1, ESK86500.1, XP_002390217.1, ESK84070.1, EUC56157.1, EMR63978.1, XP_001225528.1, EHL03569.1, XP_006692297.1, CCF32856.1, XP_003708922.1, CCO30289.1, EIN14756.1, EPE34924.1, XP_002392498.1, EJD06723.1, EPE27705.1, XP_001224385.1, XP_001840230.2, XP_001887796.1, EGY23318.1, CCA68244.1, XP_003298432.1, CCO29794.1, XP_001939876.1, XP_003300728.1, CCO36789.1, EJD03600.1, XP_003001381.1, XP_002393652.1, XP_003000738.1, CCA76671.1, EPE34273.1, XP_001272584.1, XP_003031503.1, EMD96317.1, XP_001832633.2, XP_002392615.1, XP_001906532.1, EHL00249.1, ABV46587.1, ENH87602.1, XP_001830356.2, XP_003044387.1, CCX12196.1, XP_003008052.1, XP_003002930.1, EMT71907.1, XP_003002068.1, AEO59482.1, EHK96559.1, XP_003299671.1, XP_001793524.1, EJT76904.1, XP_001806210.1, XP_003027866.1, XP_001880264.1, XP_001802359.1, EKG21252.1, EGX50459.1, XP_001880265.1, EMR63285.1, EMR66080.1, CCF37060.1, CCA66803.1, XP_003029597.1, ESU08037.1, EMR62671.1, CCO32214.1, CBY00196.1, EKG21641.1, CCO33738.1, XP_002389015.1, XP_003008244.1, EPS41803.1, EPS96370.1, CCD45436.1, EMR86091.1, EUC60658.1, CCO33588.1, XP_002476952.1, XP_001406958.1, EMR66139.1, XP_001796133.1, XP_001222239.1, XP_001227508.1, XP_001887994.1, XP_002391588.1, EOO03590.1, YP_001260935.1, EJU01358.1, XP_002911925.1, CCO38172.1, EHL00117.1, XP_001226220.1, EHK99527.1, EQB47906.1, EJF61759.1, EIW56125.1, XP_001229971.1, CCO32854.1, CCO33230.1, EQB54844.1, XP_001910397.1, XP_002388995.1, EHK98703.1, XP_001883971.1, XP_001796313.1, XP_003661743.1, EOA81556.1, XP_002392372.1, XP_001560894.1, EJF62112.1, XP_003709033.1, EOO03738.1, XP_001226548.1, CCO34834.1, CCF43136.1, EMR61290.1, ELQ42200.1, XP_003306203.1, EMR72485.1, EUC57402.1, EOO01719.1, XP_002392321.1, EKG11102.1, CCO37441.1, EIW82545.1, XP_002389005.1, EHK97736.1, XP_001227602.1, CCP37672.1, EUC55333.1, EGS17840.1, XP_370106.2, ELR02959.1, XP_002399025.1, XP_002398849.1, CCA76320.1, ELR09325.1, CCP37668.1, CCP37675.1, CCO29791.1, CCF43564.1, CCP37678.1, XP_001791239.1, CBY01256.1, XP_003328071.2, CCO32373.1, CCP37666.1, EGO55637.1, CCP37667.1, CCP37676.1, CCP37673.1, XP_002386478.1, XP_001877259.1, CCO27434.1, CBX91886.1, CCP37674.1, EUN24018.1, XP_001882292.1, XP_003650262.1, EDR07117.1, EUC60759.1, XP_002386746.1, CCF32531.1, XP_002386353.1, CCP37669.1, XP_002910575.1, CCP37670.1, XP_001881959.1, CCF42888.1, XP_002391587.1

**GenBank accession numbers of protein sequences included in AA10exp.**

XP_005802700.1, XP_001318879.1, XP_006178701.1, XP_006197692.1, EPY85497.1, XP_006197693.1, XP_006178702.1, WP_018013849.1, YP_003075103.1, XP_002674498.1, WP_018014563.1, YP_003073989.1, WP_019605052.1, WP_018275256.1, WP_018416855.1, WP_019601586.1, WP_017365650.1, WP_007075982.1, YP_004855285.1, WP_019600826.1, YP_113950.1, WP_018414756.1, WP_018275544.1, YP_003074696.1, WP_018014162.1, WP_019602152.1, WP_019604123.1, YP_001982938.1, YP_003074283.1, ABS72374.1, WP_018417086.1, WP_018015786.1, YP_004601736.1, YP_003635135.1, WP_016188914.1, YP_290015.1, WP_006142913.1, YP_003338211.1, NP_630627.1, WP_023588550.1, NP_823031.1, WP_003972433.1, WP_007388978.1, WP_019328698.1, WP_003994158.1, WP_009717693.1, YP_004404308.1, YP_007520393.1, WP_004980965.1, WP_004922771.1, WP_003996486.1, AEM44250.1, XP_004359553.1, WP_006381358.1, WP_010358032.1, WP_020125623.1, WP_009343816.1, YP_006248638.1, YP_006882144.1, WP_017626189.1, WP_009191509.1, WP_017538244.1, WP_018830962.1, WP_007072366.1, YP_001159773.1, WP_018740655.1, WP_018833033.1, WP_007457075.1, WP_019062868.1, WP_018216126.1, WP_019868685.1, WP_018251451.1, WP_018223511.1, WP_018738941.1, WP_018814167.1, WP_018828627.1, WP_018745393.1, WP_018732314.1, WP_010045973.1, YP_001982933.1, WP_020272372.1, , WP_018811296.1, YP_008792586.1, YP_004965152.1, YP_004081611.1, YP_003834803.1, WP_018788462.1, WP_018014326.1, WP_019985188.1, YP_003487398.1, WP_019603492.1, WP_005482478.1, WP_020119192.1, YP_003074927.1, WP_018415736.1, WP_007457501.1, WP_018275443.1, WP_019604285.1, WP_016823831.1, YP_001544906.1, YP_004085313.1, YP_003835802.1, YP_007950942.1, YP_003160698.1, AAF22274.1, WP_020525082.1, YP_004801042.1, YP_289670.1, WP_019521732.1, AGL12186.1, WP_007463553.1, YP_004602193.1, YP_003767344.1, YP_004453430.1, ETK36083.1, YP_003099585.1, WP_018553391.1, YP_004451583.1, WP_018105832.1, XP_002144752.1, WP_017563285.1, XP_002340725.1, YP_003683447.1, WP_019609331.1, YP_003325742.1, WP_017585858.1, WP_018523520.1, YP_004926616.1, WP_003041990.1, WP_017569469.1, WP_017581421.1, EFA84504.1, WP_005310756.1, YP_005823626.1, WP_018013554.1, YP_003072083.1, WP_017574071.1, WP_019601273.1, YP_003680374.1, WP_018275634.1, WP_019603842.1, WP_004980969.1, YP_001676932.1, WP_023588552.1, WP_006381356.1, WP_007385901.1, WP_003084461.1, YP_005825529.1, WP_019071366.1, WP_009343830.1, WP_020119191.1, YP_003487399.1, WP_004922772.1, WP_018534295.1, WP_003996487.1, WP_003994157.1, WP_020125622.1, WP_020133282.1, YP_007520394.1, WP_016823832.1, YP_006248637.1, ADX41579.1, WP_020543723.1, WP_006130848.1, WP_019604719.1, WP_018016164.1, WP_018417088.1, WP_019603300.1, YP_003072277.1, WP_019605720.1, YP_003074281.1, YP_003103005.1, WP_018277081.1, WP_018414401.1, WP_020272371.1, WP_010045975.1, WP_004288110.1, YP_004453442.1, YP_006265545.1, YP_007857878.1, WP_019603531.1, YP_898274.1, WP_018784937.1, YP_004081973.1, YP_003835309.1, WP_007642351.1, WP_019611177.1, WP_019992865.1, YP_003682451.1, WP_018015784.1, ACR23658.1, WP_003033410.1, WP_017585730.1, WP_018277782.1, WP_017579126.1, WP_017571088.1, YP_003638186.1, WP_020141309.1, WP_016189517.1, YP_007953654.1, YP_001543076.1, YP_003161093.1, WP_020543321.1, YP_004908734.1, YP_004909108.1, YP_001278902.1, WP_007262244.1, YP_003638629.1, YP_003074282.1, YP_001545412.1, WP_022013656.1, AGN70390.1, WP_009720086.1, WP_018552941.1, YP_004967448.1, YP_005463250.1, ETK32597.1, WP_019603530.1, YP_003636134.1, WP_018105029.1, YP_004600776.1, WP_020522078.1, YP_003115663.1, YP_001047116.1, YP_003638634.1, ETK37505.1, YP_003638125.1, YP_001546226.1, WP_010522514.1, WP_018015785.1, WP_010179473.1, YP_004406959.1, WP_007457316.1, YP_003637118.1, WP_007462836.1, WP_019604720.1, WP_018277781.1, ETK31510.1, CAA43597.1, ADK13057.1, YP_001981949.1, YP_003678873.1, WP_017572681.1, YP_003099662.1, WP_007079530.1, YP_003652627.1, WP_020517237.1, WP_018417087.1, WP_006520207.1, WP_007071284.1, WP_019071818.1, WP_018013388.1, WP_018823840.1, YP_003071836.1, WP_018276163.1, WP_023591038.1, YP_003114862.1, XP_004341251.1, WP_019601420.1, WP_020519643.1, WP_019073103.1, YP_003833867.1, YP_004080662.1, YP_003636669.1, YP_003072082.1, YP_001982934.1, ETK30551.1, YP_001543104.1, YP_004405511.1, YP_003636013.1, YP_003115658.1, WP_019602293.1, YP_003073201.1, WP_018015082.1, WP_018015083.1, WP_019602292.1, YP_003073203.1, WP_018277126.1, WP_018415105.1, WP_018415104.1, WP_019604871.1, YP_005466418.1, WP_019604870.1, WP_018277125.1, YP_003100560.1, YP_003113546.1, YP_871941.1, WP_006812005.1, WP_003862568.1, YP_006479312.1, WP_007909674.1, YP_004963776.1, YP_007951267.1, WP_007076471.1, YP_003614872.1, WP_023620525.1, YP_001984214.1, WP_020508953.1, WP_020516020.1, YP_871940.1, YP_003325739.1, EUM07739.1, ACV09037.1, YP_003074708.1, YP_003073756.1, YP_003636992.1, WP_020518411.1, YP_008734316.1, AEI35198.1, YP_008734860.1, WP_018014572.1, YP_003073972.1, YP_001547564.1, YP_003160702.1, WP_019601571.1, YP_528108.1, WP_020210924.1, YP_528403.1, WP_019603529.1, ETK34775.1, YP_001982932.1, CAA33469.1, WP_004287216.1, YP_001543074.1, WP_003064656.1, ETK31548.1, YP_001983791.1, WP_007459135.1, YP_005824290.1, YP_898832.1, AHH46736.1, YP_001121639.1, WP_003036972.1, YP_005826149.1, WP_004339565.1, WP_003034334.1, YP_004810442.1, AEI36060.1, YP_001676833.1, YP_004081741.1, WP_004287998.1, YP_003102358.1, AGU68247.1, WP_007643892.1, YP_003767356.1, YP_006268300.1, WP_020512002.1, WP_017589825.1, YP_003680375.1, WP_017582185.1, YP_001983071.1, YP_005465209.1, YP_003681426.1, WP_019605211.1, WP_018277482.1, YP_004800724.1, WP_003032333.1, YP_001982936.1, WP_022737925.1, EUM07741.1, WP_017566121.1, YP_003160701.1, WP_018015396.1, YP_003075599.1, GAE63198.1, WP_019328699.1, WP_007074234.1, ACQ89878.1, WP_007638349.1, ABI83194.1, YP_003835082.1, CAJ79847.1, YP_004085009.1, WP_018277780.1, WP_019121191.1, AFX71044.1, CAA60493.1, YP_001983438.1, WP_007071583.1, WP_020509871.1, WP_017571419.1, YP_008736267.1, YP_004802777.1, YP_525540.1, WP_017541868.1, WP_017557665.1, ETA71039.1, YP_526124.1, YP_004115963.1, WP_021183078.1, WP_005475300.1, ACY75514.1, YP_432126.1, WP_018105644.1, ETK31387.1, WP_005474921.1, YP_007523552.1, WP_017624266.1, WP_018691604.1, BAM62868.1, WP_004934806.1, YP_003638250.1, WP_010048734.1, WP_023537669.1, WP_003988311.1, YP_527176.1, WP_006230078.1, WP_017099707.1, WP_017102181.1, YP_002417302.1, YP_008791331.1, YP_004081489.1, YP_003834680.1, WP_020126037.1, WP_007075371.1, YP_001983448.1, WP_010389311.1, WP_023398099.1, YP_006916186.1, ETK32165.1, NP_668292.1, NP_992095.1, YP_005509883.1, YP_005625272.1, YP_004752932.1, WP_004003745.1, YP_006267062.1, WP_018749669.1, WP_016191604.1, WP_017023869.1, WP_017800101.1, YP_005464722.1, WP_016900118.1, WP_016706401.1, YP_004065202.1, AGU01017.1, WP_010362143.1, WP_010386091.1, WP_006217774.1, WP_020140407.1, WP_009838792.1, YP_005531199.1, WP_017902400.1, AAC79666.1, WP_017130002.1, WP_010559776.1, AAT81213.1, AAP49340.1, WP_010863923.1, WP_010377869.1, WP_010607422.1, WP_020210245.1, WP_017016532.1, WP_017011427.1, WP_000730724.1, WP_017006819.1, WP_017016045.1, WP_017004429.1, WP_017014236.1, WP_017009312.1, WP_016960083.1, WP_002541841.1, WP_002537929.1, WP_017003740.1, WP_017006052.1, WP_017218240.1, WP_000728624.1, WP_016400332.1, YP_003114337.1, YP_003487624.1, YP_004117159.1, WP_021222437.1, WP_001908705.1, YP_002876271.1, WP_001907850.1, WP_001910343.1, WP_005482075.1, YP_001980898.1, EUJ35680.1, WP_005446095.1, YP_006917849.1, YP_004452793.1, WP_007645716.1, YP_004407488.1, WP_003176556.1, WP_005528284.1, YP_526130.1, WP_010761983.1, WP_010647647.1, WP_010770365.1, WP_009306938.1, WP_007459053.1, WP_019981836.1, WP_006230306.1, WP_005442917.1, EUM08171.1, WP_003993916.1, WP_007463386.1, WP_008351042.1, WP_010763586.1, WP_020275437.1, WP_023545831.1, WP_009706005.1, WP_004981713.1, WP_019063778.1, WP_022940971.1, WP_002374219.1, WP_006960811.1, WP_019276424.1, WP_020641306.1, WP_016400467.1, WP_020125388.1, WP_018571551.1, YP_008389393.1, WP_020515768.1, YP_003770242.1, WP_010448212.1, WP_006136572.1, YP_003113484.1, WP_007073763.1, YP_004453273.1, YP_003014805.1, WP_020197353.1, YP_008935325.1, WP_019071560.1, WP_003156509.1, WP_017782255.1, WP_019612759.1, WP_009050797.1, WP_017780423.1, ABU71153.1, WP_017041345.1, YP_001346803.1, WP_007928332.1, WP_017765254.1, WP_005427638.1, WP_004874039.1, WP_017784627.1, WP_016703584.1, WP_017039795.1, WP_023967404.1, WP_009045569.1, WP_006142896.1, EKM29260.1, YP_006248360.1, WP_017787469.1, WP_018547112.1, WP_004722689.1, WP_007462097.1, WP_017800519.1, WP_003996811.1, WP_010042447.1, WP_017240824.1, WP_009841677.1, ADK32573.1, WP_016431882.1, WP_019830309.1, WP_007724611.1, WP_019275094.1, YP_855149.1, WP_017408975.1, WP_016739581.1, YP_002775165.1, WP_017252583.1, WP_021455904.1, YP_001007736.1, YP_003111267.1, WP_006960889.1, WP_004923270.1, WP_006030399.1, WP_001913569.1, WP_001959513.1, WP_010351055.1, WP_006217776.1, YP_007520677.1, YP_006386197.1, WP_017019333.1, WP_020639945.1, YP_003101875.1, YP_206101.1, WP_016792410.1, YP_002157735.1, WP_010762341.1, WP_010772090.1, WP_017037056.1, WP_017022618.1, CAQ80888.1, NP_716699.1, WP_009697450.1, AFN30403.1, YP_737003.1, WP_017033507.1, YP_735155.1, WP_007548887.1, WP_007475382.1, WP_008045076.1, YP_003637994.1, WP_023602659.1, WP_005421954.1, WP_010565979.1, WP_021710477.1, WP_020118950.1, WP_017066164.1, WP_017065526.1, WP_017059161.1, WP_020133604.1, WP_003335212.1, WP_009601339.1, WP_018671414.1, WP_022584557.1, WP_009837614.1, EUB33448.1, WP_020413224.1, YP_003637596.1, WP_017630513.1, WP_008047870.1, WP_007968318.1, YP_005801550.1, WP_004742391.1, WP_008025185.1, YP_005205864.1, WP_017082465.1, WP_017070909.1, WP_004730095.1, YP_131985.1, WP_004741633.1, WP_003186601.1, WP_017106236.1, WP_008041848.1, WP_005474669.1, WP_020796159.1, WP_017061750.1, WP_017093071.1, WP_008217215.1, WP_007975604.1, WP_019820266.1, WP_017075828.1, WP_017064832.1, WP_017094667.1, WP_017085432.1, WP_017079651.1, WP_004734903.1, WP_017088639.1, WP_007989333.1, YP_003160700.1, YP_003652167.1, WP_009652316.1, WP_004098112.1, WP_005484218.1, YP_001906724.1, WP_023584501.1, WP_005397801.1, WP_021450529.1, YP_008270597.1, ETZ08868.1, NP_801108.1, YP_008548196.1, YP_005816909.1, YP_007301490.1, YP_001446875.1, WP_005374894.1, WP_006879305.1, WP_017447203.1, WP_005458314.1, ETJ86532.1, WP_005499929.1, WP_021822056.1, YP_008267827.1, WP_008078398.1, WP_005384596.1, ETK28080.1, ETT16428.1, WP_004412150.1, WP_021484027.1, WP_017634444.1, YP_003288274.1, YP_004909133.1, YP_773321.1, YP_008930063.1, WP_006760918.1, WP_006710362.1, NP_631677.1, YP_001920879.1, WP_017738435.1, WP_003372857.1, WP_009387951.1, WP_009846927.1, WP_000976431.1, WP_010052631.1, YP_006994094.1, WP_008217555.1, WP_005520720.1, YP_003073663.1, YP_003074107.1, WP_000744632.1, WP_022579053.1, WP_001041518.1, WP_005594090.1, WP_002045306.1, WP_000744641.1, WP_000744640.1, WP_005525611.1, WP_001898077.1, WP_001041519.1, WP_001964687.1, NP_762031.1, WP_023268383.1, WP_006765932.1, WP_001893514.1, YP_001215264.1, WP_000744639.1, YP_004190719.1, YP_002812387.1, YP_007015995.1, NP_936607.1, WP_017422524.1, WP_004748754.1, WP_001888865.1, WP_000744633.1, WP_000744643.1, YP_001140518.1, WP_017790749.1, WP_021257881.1, WP_004128259.1, WP_017057421.1, WP_004108915.1, WP_021255139.1, WP_004117536.1, WP_023320224.1, YP_003611175.1, WP_016809081.1, AFM58264.1, WP_010441194.1, P07986.1, WP_023299191.1, EUM08209.1, WP_023331899.1, WP_023305330.1, WP_023336700.1, XP_004151141.1, WP_023656051.1, EUM58780.1, WP_022646879.1, WP_006069139.1, YP_006577022.1, WP_008217553.1, YP_004600127.1, YP_006268132.1, WP_007914528.1, WP_004559598.1, EUM53187.1, YP_435117.1, EUM17042.1, WP_017383905.1, WP_019362105.1, YP_007847581.1, EUM21351.1, WP_006810316.1, EUM43405.1, EUL91028.1, WP_023295823.1, WP_016798730.1, WP_022650291.1, WP_023315294.1, EUM48107.1, WP_023302987.1, EUM83239.1, WP_016799756.1, EUM25366.1, WP_003863387.1, WP_017694635.1, EUL77000.1, ABH79487.1, WP_010452049.1, WP_004734902.1, WP_010453660.1, YP_001049290.1, WP_019360898.1, YP_006021911.1, YP_562041.1, YP_001367656.1, WP_006083963.1, YP_002359297.1, ADT95748.1, YP_007953655.1, WP_016710875.1, WP_004170697.1, ADK32576.1, WP_017068277.1, ETK36906.1, YP_003537799.1, ADE37527.1, EUJ46126.1, YP_850613.1, YP_006682921.1, YP_002349108.1, YP_006674277.1, WP_010381259.1, NP_471941.1, YP_002759122.1, YP_008287095.1, WP_003768277.1, WP_021496618.1, YP_006680043.1, YP_006691557.1, WP_003772369.1, WP_010367339.1, WP_003727915.1, YP_008273927.1, WP_003739730.1, YP_008295550.1, EFR86758.1, NP_465990.1, YP_007604914.1, YP_005963574.1, YP_005966465.1, YP_001762232.1, YP_003414770.1, WP_023399202.1, WP_008482126.1, ETX40784.1, BAO32502.1, YP_007951167.1, ETX49935.1, WP_010605520.1, Q8EHY2.2, YP_007346214.1, WP_010378666.1, WP_017216868.1, EUJ46507.1, WP_010862264.1, WP_010635191.1, YP_008041757.1, WP_019840850.1, AHE48177.1, WP_005332396.1, WP_021138864.1, YP_003652743.1, WP_005313702.1, WP_005308580.1, WP_005357861.1, WP_004373688.1, WP_005340269.1, WP_006323970.1, YP_008157891.1, WP_005495563.1, YP_004394209.1, WP_005343068.1, WP_006322254.1, WP_010672829.1, WP_019439553.1, YP_001189587.1, WP_021230904.1, WP_007457174.1, YP_004965560.1, WP_017222644.1, WP_004958531.1, WP_024063585.1, YP_006267701.1, WP_017894022.1, YP_001480461.1, WP_005352628.1, WP_017336131.1, WP_021783683.1, ACM41799.1, YP_003636526.1, ACC90457.1, WP_018015605.1, WP_016613730.1, WP_005233090.1, WP_016608548.1, CAH22604.1, WP_020410109.1, WP_016621983.1, YP_003637995.1, YP_008597426.1, YP_003162169.1, WP_009684961.1, WP_003339108.1, WP_003303830.1, ETK31828.1, WP_017903692.1, Q01786.2, YP_288962.1, WP_020646728.1, YP_001719446.1, WP_010736570.1, WP_018603489.1, WP_017130065.1, WP_010090092.1, YP_008823129.1, WP_019722405.1, YP_001399602.1, YP_007949771.1, AHF79328.1, WP_003307509.1, YP_006265968.1, WP_007456826.1, WP_023934569.1, WP_008988865.1, WP_017071808.1, WP_017068473.1, WP_018788190.1, AFK65733.1, XP_641359.1, WP_018523036.1, YP_003834839.1, YP_004081647.1, WP_007071439.1, YP_005465017.1, WP_010053940.1, WP_008381854.1, YP_007754247.1, WP_010748912.1, YP_003651542.1, WP_020510659.1, WP_020584075.1, WP_017152765.1, YP_001375457.1, YP_003327705.1, BAJ21448.1, YP_004404351.1, WP_001049447.1, WP_001049728.1, WP_000769027.1, WP_002016996.1, YP_006828541.1, YP_008820164.1, WP_016134520.1, YP_005573405.1, WP_001049446.1, WP_001049837.1, WP_001049844.1, WP_000769022.1, WP_001049839.1, WP_001049841.1, YP_006601928.1, WP_016130657.1, WP_001049842.1, YP_002446999.1, YP_001645189.1, WP_016098782.1, WP_001049445.1, WP_001049832.1, WP_002147840.1, WP_002127483.1, WP_002168344.1, WP_016120232.1, WP_001049845.1, WP_002085456.1, AHG19437.1, WP_016101970.1, WP_002065509.1, WP_001049833.1, WP_016087815.1, WP_001049836.1, WP_001049834.1, WP_016077406.1, WP_002032213.1, YP_007422859.1, WP_017657788.1, WP_001049843.1, WP_016080749.1, WP_001049730.1, WP_001049727.1, WP_002136433.1, WP_016095358.1, WP_000769025.1, YP_003765393.1, WP_002195554.1, WP_002061559.1, WP_001060176.1, WP_005364129.1, WP_006644242.1, WP_002197854.1, WP_001034445.1, WP_001034446.1, WP_001034447.1, WP_016623638.1, YP_003766420.1, WP_018014068.1, YP_008734315.1, WP_016096868.1, WP_010827776.1, WP_005230510.1, WP_002371718.1, WP_002425284.1, YP_008823130.1, ACJ82852.1, WP_005517847.1, YP_245766.1, WP_001035087.1, WP_001035090.1, YP_002533312.1, WP_001035088.1, WP_002137415.1, WP_001035096.1, WP_001035086.1, WP_002069392.1, WP_002016535.1, P26414.1, YP_003653422.1, WP_020137104.1, AHH97713.1, YP_003836725.1, YP_004405633.1, WP_001065148.1, WP_001065160.1, WP_002189836.1, WP_001065138.1, WP_001065149.1, YP_008819434.1, YP_007422288.1, WP_001065143.1, WP_001065161.1, YP_005572751.1, WP_000795734.1, YP_002367511.1, WP_001065166.1, WP_016081302.1, WP_001065159.1, WP_001065158.1, WP_001991542.1, YP_895304.1, WP_001065139.1, YP_005119371.1, WP_001065243.1, WP_001065152.1, YP_006598270.1, YP_002338795.1, YP_003665034.1, WP_016134690.1, NP_979128.1, WP_016512687.1, YP_002446230.1, WP_001992383.1, WP_001065252.1, WP_000795733.1, YP_005566440.1, WP_000795731.1, WP_000795728.1, WP_006714574.1, YP_001645428.1, WP_001065140.1, WP_001065162.1, WP_002168229.1, WP_001065135.1, WP_002171876.1, WP_001065163.1, WP_002329865.1, WP_016122655.1, WP_002142062.1, YP_006602605.1, WP_001065245.1, WP_001088483.1, WP_016127104.1, WP_002165869.1, WP_001065147.1, WP_016101279.1, WP_002127733.1, WP_016120338.1, WP_001065145.1, WP_001065150.1, WP_002013028.1, ACW83015.1, WP_016091128.1, YP_008780779.1, WP_016095577.1, YP_084112.1, WP_016091524.1, WP_002065809.1, YP_005462041.1, WP_016087558.1, WP_002085112.1, YP_003792526.1, YP_001645018.1, WP_002136717.1, YP_006610416.1, WP_001065153.1, WP_001065215.1, WP_001065136.1, WP_016110132.1, WP_002032659.1, WP_002194809.1, WP_001065251.1, WP_010750601.1, WP_001065134.1, WP_000797165.1, WP_001065249.1, WP_000797164.1, YP_001544437.1, YP_003911962.1, WP_001065254.1, WP_005592969.1, YP_036882.1, YP_002451750.1, NP_845142.1, WP_003189842.1, WP_001065137.1, WP_000795727.1, WP_002148110.1, AEP40514.1, WP_016177252.1, WP_005875405.1, WP_010829191.1, YP_003635455.1, WP_018099985.1, WP_007129910.1, WP_009591351.1, YP_003242381.1, YP_004084402.1, YP_288961.1, WP_008177368.1, WP_020515925.1, WP_010816441.1, YP_005463778.1, WP_000589789.1, AHF79236.1, YP_006265110.1, WP_002001856.1, WP_010859043.1, WP_010735559.1, WP_006179087.1, WP_003284869.1, WP_003310495.1, WP_009673860.1, WP_003279245.1, WP_003303839.1, WP_017605930.1, WP_002063099.1, WP_019723901.1, ETK36923.1, WP_006766319.1, WP_003291850.1, WP_005549632.1, YP_440119.1, WP_021255070.1, WP_021260409.1, WP_021261650.1, WP_016392732.1, WP_006675569.1, WP_009222736.1, WP_016312786.1, WP_019419323.1, YP_003101853.1, WP_018274594.1, ETT40072.1, WP_019722404.1, ETT42610.1, WP_006210296.1, WP_009673792.1, WP_021253357.1, WP_010498212.1, WP_006210199.1, YP_004500686.1, DAA01337.1, YP_003341864.1, WP_021259864.1, WP_021262861.1, YP_006915022.1, WP_021252423.1, YP_003074615.1, YP_003635360.1, WP_019422163.1, WP_005549180.1, WP_006677739.1, WP_021255665.1, WP_000224645.1, ETK33887.1, YP_003160505.1, WP_019606295.1, WP_018414832.1, EUJ35055.1, YP_008138305.1, YP_003313237.1, YP_006828806.1, ETK38080.1, WP_019602218.1, YP_005017821.1, WP_020646998.1, WP_002043841.1, AFQ34194.1, WP_006381528.1, AFQ34195.1, WP_023483239.1, YP_008967963.1, YP_003327043.1, WP_018417286.1, WP_017582312.1, YP_004601785.1, YP_289135.1, YP_008824171.1, WP_020521856.1, YP_003638201.1, AAZ55700.1, WP_020511890.1, WP_018275241.1, YP_003651397.1, ETK38093.1, YP_003835084.1, WP_022943491.1, WP_016623522.1, WP_023621422.1, WP_002632296.1, WP_020542391.1, YP_004451934.1, WP_010089587.1, ETK35711.1, WP_020523700.1, YP_003099616.1, YP_005049715.1, YP_005464857.1, YP_001698788.1, ETK37492.1, YP_007950812.1, WP_016188476.1, YP_003101672.1, YP_003653227.1, YP_004081743.1, WP_020516019.1, YP_007951268.1, WP_003278596.1, WP_017044239.1, WP_017045958.1, WP_017050280.1, YP_004577742.1, YP_337198.1, YP_006657568.1, WP_007455959.1, YP_004084230.1, YP_003836913.1, WP_007073485.1, XP_004359308.1, YP_006265974.1, YP_004093638.1, WP_017111782.1, WP_006676405.1, WP_008224296.1, WP_019419277.1, WP_006038041.1, WP_004538030.1, WP_020582299.1, WP_004525523.1, YP_001061758.1, WP_004549547.1, WP_004530016.1, WP_019817448.1, YP_006326344.1, WP_020302142.1, WP_007457337.1, WP_019256488.1, YP_001761089.1, WP_019185268.1, WP_001898032.1, WP_001891871.1, WP_001904547.1, NP_232540.1, WP_003450218.1, WP_001893818.1, WP_003451861.1, WP_001885116.1, WP_009507194.1, NP_936142.1, WP_017791435.1, WP_008899396.1, WP_017421745.1, NP_763162.2, YP_006245335.1, YP_003635293.1, WP_007461359.1, ETZ12006.1, YP_008967279.1, WP_023484147.1, ACE83992.1, WP_007638856.1, WP_017066081.1, WP_001900391.1, YP_005463772.1, YP_005184587.1, WP_017562452.1, WP_007073583.1, YP_002264569.1, WP_017021022.1, WP_023602761.1, ABZ87418.1, WP_021706310.1, YP_004081719.1, WP_019276683.1, WP_006959135.1, YP_003177200.1, YP_003650975.1, WP_005434826.1, ADV88087.1, WP_021456945.1, YP_008548409.1, WP_010453448.1, WP_005447299.1, WP_005497737.1, YP_008268052.1, WP_005428273.1, WP_010645424.1, YP_003288058.1, WP_017633838.1, WP_023584373.1, WP_009842223.1, WP_005533118.1, WP_023623110.1, ETO49972.1, WP_009706787.1, WP_005383277.1, WP_005460775.1, WP_021452792.1, WP_005391973.1, WP_021710686.1, ETT16213.1, YP_008270825.1, WP_017447048.1, WP_021484189.1, WP_005374361.1, WP_020195107.1, WP_005487067.1, WP_021450382.1, YP_007300114.1, NP_799602.1, YP_002157582.1, WP_017818359.1, YP_205971.1, WP_005421689.1, WP_017019968.1, WP_010317603.1, YP_003835060.1, YP_005367520.1, NP_823430.1, YP_008620535.1, YP_004794463.1, WP_022598204.1, ACF53577.1, WP_022604506.1, WP_022614024.1, WP_005474084.1, WP_004412448.1, WP_004746366.1, WP_005414838.1, WP_021203315.1, YP_002030259.1, YP_001974152.1, WP_019661678.1, WP_021203314.1, WP_005411564.1, WP_019336748.1, WP_008267196.1, AAP42509.1, WP_018555375.1, WP_017686339.1, WP_000873598.1, WP_000873596.1, WP_000873597.1, WP_019829514.1, WP_000873601.1, WP_000873603.1, WP_000873604.1, WP_022578664.1, WP_000873582.1, WP_020329759.1, WP_017035907.1, AEA79770.1, WP_000873602.1, WP_000873586.1, WP_002156693.1, AGU11296.1, YP_006186729.1, WP_005411565.1, YP_004794462.1, WP_000873594.1, YP_006186728.1, WP_000873580.1, WP_005420219.1, WP_010482246.1, WP_001965441.1, WP_023084930.1, WP_023112301.1, WP_000873588.1, WP_000873593.1, WP_000873591.1, AAF12807.1, NP_249543.1, WP_002043266.1, WP_000873590.1, WP_019725852.1, WP_003163183.1, WP_003120756.1, AGV66685.1, WP_023130171.1, YP_792425.1, YP_008131285.1, WP_009876003.1, YP_007711058.1, WP_000873579.1, YP_006484258.1, WP_023129084.1, YP_005979876.1, WP_003122990.1, CDH72609.1, WP_000873595.1, YP_003162382.1, WP_000873600.1, WP_002052805.1, WP_000873583.1, WP_008355751.1, WP_019396342.1, YP_001350011.1, WP_005420221.1, WP_017656464.1, ETW99446.1, WP_004396884.1, CCP10740.1, CAA54706.1, WP_019661679.1, WP_002071993.1, WP_019336749.1, YP_001974153.1, WP_020393078.1, WP_010482247.1, AAQ60262.1, WP_007388159.1, WP_004807686.1, WP_004653135.1, WP_005316068.1, WP_016163457.1, WP_005293408.1, WP_020299895.1, WP_005211932.1, WP_005420222.1, YP_003834462.1, YP_004081257.1, WP_005152408.1, WP_005203185.1, WP_005241108.1, WP_005189230.1, WP_016652594.1, WP_009088928.1, WP_020478591.1, WP_023269957.1, WP_002639210.1, WP_005271000.1, WP_016540404.1, WP_007462590.1, EKM29767.1, WP_002619418.1, WP_007456986.1, NP_902993.1, YP_003834284.1, WP_019527349.1, WP_019986217.1, NP_900224.1, WP_005320876.1, WP_019185455.1, WP_021475839.1, YP_004800964.1, YP_001024877.1, WP_018788380.1, WP_004530781.1, WP_019724455.1, CCD07519.1, WP_006142774.1, WP_020126401.1, WP_018786693.1, ETK35491.1, WP_020517503.1, WP_004727165.1, YP_003837344.1, YP_004083709.1, WP_008265948.1, NP_821730.1, WP_007456093.1, YP_006243043.1, YP_006267884.1, ADJ46951.1, WP_020517238.1, BAA25629.1, YP_008735113.1, YP_004403782.1, ADG87762.1, YP_004643617.1, YP_008789286.1, NP_932669.1, WP_018552318.1, YP_008734907.1, NP_787918.1, YP_004812344.1, WP_007075026.1, YP_007952241.1, YP_003635433.1, WP_006373980.1, ADK32572.1, YP_007520930.1, YP_005463408.1, WP_009714393.1, WP_018099987.1, WP_010118051.1, YP_003834761.1, YP_007929051.1, WP_010108136.1, YP_004081568.1, YP_004907660.1, YP_008378590.1, YP_291046.1, NP_848371.1, WP_009933012.1, WP_006029789.1, YP_007921760.1, YP_004406840.1, WP_005310552.1, AHE35282.1, WP_020519912.1, ETK30874.1, WP_018487974.1, WP_003977643.1, NP_625478.1, YP_004927228.1, YP_008992182.1, CAJ90160.1, YP_007857171.1, YP_006243046.1, YP_611057.1, WP_019327463.1, WP_007388160.1, YP_007520618.1, ETK35682.1, YP_007953380.1, WP_007072392.1, YP_003635290.1, YP_004600962.1, AEN11025.1, YP_004404263.1, WP_020517264.1, WP_019527348.1, WP_010353132.1, WP_004923736.1, AFY62890.1, YP_008378439.1, WP_017582181.1, WP_004561891.1, WP_018251522.1, YP_005533220.1, WP_001926535.1, WP_004979254.1, WP_018223425.1, YP_001821711.1, WP_018833083.1, WP_003964065.1, YP_004961303.1, WP_018814129.1, WP_018731704.1, WP_018722933.1, WP_018740699.1, YP_001159852.1, WP_018216208.1, WP_004921375.1, WP_018570430.1, YP_003342987.1, WP_007201237.1, YP_007862506.1, YP_004922206.1, ETK38143.1, WP_006141838.1, WP_017605929.1, WP_007076514.1, WP_019032906.1, WP_018795250.1, WP_007071991.1, WP_018802912.1, YP_001538057.1, WP_018789303.1, WP_019900823.1, WP_018798826.1, WP_018824238.1, WP_018908454.1, WP_016812939.1, WP_019325321.1, WP_018893019.1, WP_018804481.1, AAD27623.1, WP_018586666.1, WP_018801344.1, CAE53336.1, NP_203226.1, WP_018515450.1, YP_003487786.1, YP_006265547.1, WP_007447920.1, AFO53541.1, WP_020510327.1, WP_007499849.1, YP_004816463.1, WP_018487975.1, WP_019610726.1, WP_017543571.1, WP_017945331.1, WP_020522187.1, YP_001158490.1, NP_626007.1, YP_004084095.1, WP_003949941.1, AGL17244.1, YP_004451802.1, YP_003837053.1, YP_005464841.1, WP_003993703.1, WP_017589822.1, WP_020125198.1, WP_007385369.1, WP_019983661.1, YP_003680372.1, YP_007930129.1, WP_017592328.1, WP_020514762.1, WP_020543319.1, WP_009672322.1, WP_006376680.1, YP_008793902.1, YP_003341210.1, WP_010033940.1, YP_008003691.1, WP_016325887.1, WP_003977092.1, YP_003487697.1, WP_020273736.1, YP_007951276.1, YP_008004025.1, WP_018822928.1, WP_018727378.1, WP_018811176.1, AAL01718.1, WP_005485043.1, ACZ31262.1, WP_018849101.1, Q05894.1, WP_020132575.1, WP_020544469.1, WP_017573674.1, WP_002625778.1, WP_019059242.1, WP_016826935.1, WP_020128202.1, WP_017673282.1, WP_016823264.1, EPQ29024.1, YP_007858524.1, WP_019434545.1, WP_007075442.1, WP_003997085.1, WP_005520536.1, YP_004926016.1, WP_009673940.1, WP_010070266.1, WP_006677168.1, WP_016905196.1, CAD91236.1, WP_019422273.1, WP_018523034.1, WP_018959610.1, WP_020678942.1, WP_010355839.1, WP_023542709.1, YP_001827285.1, YP_003114991.1, WP_003996940.1, WP_020122176.1, WP_020133804.1, WP_010057010.1, WP_017568593.1, YP_008385440.1, WP_008744121.1, GAC97794.1, WP_009193079.1, WP_004173206.1, WP_018744303.1, WP_019868754.1, WP_007132473.1, YP_003240889.1, WP_009590744.1, WP_006208124.1, WP_019544022.1, ETT40550.1, WP_023963184.1, WP_006131500.1, BAO37620.1, NP_827736.1, YP_004801624.1, WP_007448658.1, WP_016644916.1, WP_023589431.1, WP_004705552.1, WP_009676479.1, WP_007380986.1, WP_019066204.1, WP_003970081.1, YP_008004371.1, YP_008004671.1, WP_019522787.1, WP_020638544.1, WP_023963587.1, WP_018830511.1, AAD10322.1, YP_006876899.1, WP_018087895.1, WP_020609352.1, WP_021599730.1, WP_018908332.1, WP_018806490.1, WP_018588184.1, WP_018789547.1, WP_018802810.1, YP_001536515.1, WP_019031842.1, WP_018795064.1, WP_018805138.1, CBA72252.1, YP_001983593.1, WP_005481967.1, AGE89883.1, WP_004002408.1, WP_006607728.1, WP_003987193.1, WP_003989226.1, WP_019355346.1, WP_023420127.1, WP_018853987.1, WP_019073916.1, WP_018834457.1, WP_016468016.1, WP_021023489.1, WP_019602454.1, WP_020117830.1, WP_020139375.1, WP_018532408.1, WP_016578906.1, WP_005481839.1, WP_010046142.1, AAW49884.1, YP_003071742.1, AAF72586.1, YP_007954235.1, WP_018469248.1, WP_010636773.1, WP_017241592.1, YP_007748413.1, WP_008409553.1, WP_020274847.1, WP_018788630.1, WP_019885120.1, EST08257.1, WP_018416019.1, WP_018547161.1, WP_018278100.1, WP_020520990.1, WP_007075562.1, YP_007952900.1, WP_017603176.1, WP_020517505.1, YP_003848572.1, WP_019605580.1, WP_018849283.1, XP_761586.1, WP_018808230.1, WP_018583675.1, WP_019900144.1, WP_020217966.1, WP_019030411.1, WP_019033204.1, WP_017614878.1, YP_006248282.1, WP_003959914.1, WP_005307481.1, EFA82167.1, WP_023551824.1, YP_003957509.1, WP_006350123.1, WP_016434520.1, NP_900223.1, WP_006129762.1, YP_003556556.1, WP_018794594.1, YP_007525326.1, WP_017580591.1, NP_046225.1, YP_008389314.1, WP_017800102.1, WP_004990086.1, YP_473274.1, WP_018721595.1, WP_017493332.1, YP_007360395.1, WP_007373493.1, WP_018933926.1, WP_020133705.1, WP_019631646.1, WP_016431988.1, WP_010643438.1, WP_018653572.1, WP_023541211.1, WP_008412571.1, WP_023421450.1, WP_023416505.1, WP_018825566.1, WP_019062782.1, WP_019071436.1, YP_008736394.1, WP_018800581.1, WP_018794063.1, ADG73405.1, WP_018560755.1, ETS65131.1, WP_020122074.1, GAC75488.1, WP_018828381.1, WP_020125293.1, NP_823344.1, ETK35261.1, YP_008528198.1, WP_018537109.1, WP_019063285.1, YP_004902192.1, WP_022948944.1, XP_005423021.1, WP_023127741.1, YP_001545413.1, WP_017535829.1, YP_006641991.1, WP_020519976.1, YP_004904565.1, ADD42623.1, WP_016656916.1, WP_018528657.1, YP_758531.1, BAA24259.1, CCF50234.1, NP_703054.1, WP_007455885.1, WP_018810224.1, WP_003370735.1, WP_020273184.1, WP_020552147.1, YP_007520789.1, WP_019611547.1, YP_004960038.1, WP_010353231.1, WP_018549255.1, WP_020514466.1, YP_003834177.1, YP_004080977.1, WP_018789237.1, CBQ69852.1, YP_005464942.1, AEV85992.1, YP_758332.1, YP_006880524.1, WP_017236219.1, AAB47606.1, AFN21167.1, AFN21027.1, AAC63737.1, AFN08981.1, AFN21306.1, AGX01150.1, YP_002884293.1, YP_008893728.1, YP_007250471.1, WP_018488254.1, WP_005908171.1, WP_021592865.1, WP_003435477.1, YP_008795735.1, ADU56336.1, YP_950778.1, WP_023590258.1, WP_007431950.1, YP_717594.1, YP_003343307.1, NP_691731.1, WP_021289599.1, WP_017795956.1, YP_001108428.1, WP_010346651.1, WP_003029315.1, WP_004400380.1, AAG15504.1, YP_002273981.1, NP_542682.1, AAG53801.1, AEN03982.1, WP_007074975.1, WP_019639750.1, YP_005467636.1, WP_003995588.1, WP_019375874.1, WP_010034207.1, YP_874242.1, WP_017893335.1, WP_006350848.1, WP_019432034.1, YP_001479333.1, YP_004903105.1, YP_004814948.1, WP_005319339.1, ACO53470.1, WP_003321618.1, YP_008739502.1, YP_308954.1, WP_006381336.1, AAY83998.1, WP_007454166.1, WP_009893813.1, EGH44758.1, YP_003517804.1, WP_009338429.1, YP_002332720.1, YP_006607807.1, AHC69566.1, WP_006820597.1, NP_047705.1, ABY65755.1, WP_018220379.1, AAB07702.1, NP_037785.1, WP_018745623.1, WP_018794467.1, WP_018803486.1, ADV91255.1, WP_018587114.1, WP_016817746.1, WP_018909518.1, WP_020217544.1, WP_018833107.1, WP_018805379.1, YP_001159280.1, WP_018796952.1, WP_018790785.1, WP_019032687.1, YP_001539599.1, WP_018799708.1, WP_018222118.1, WP_018828520.1, CDG72366.1, WP_018812083.1, WP_018815225.1, WP_018584650.1, WP_020579087.1, WP_017602855.1, GAE32847.1, YP_008378272.1, WP_019135259.1, NP_613120.1, NP_242169.1, YP_002332567.1, NP_689207.1, AFP95751.1, AAD45231.1, ACU39154.1, AAQ11056.1, WP_018671415.1, YP_174660.1, WP_022584558.1, WP_003335213.1, ACI28732.1, WP_017812710.1, YP_001036316.1, YP_529696.1, AEV89055.1, WP_023481944.1, XP_003288597.1, AGL21532.1, WP_018685021.1, WP_003400932.1, WP_020543241.1, WP_005764584.1, WP_020313147.1, XP_003288596.1, WP_023545902.1, WP_020514710.1, XP_004361129.1, BAL90477.1, AHH96751.1, WP_003410790.1, WP_017279323.1, WP_004558451.1, WP_019046649.1, WP_016935992.1, WP_010209203.1, CAA20076.1, YP_007040559.1, AAK70680.1, ACZ83315.1, WP_006316749.1, YP_008138004.1, YP_004362029.1, WP_018103444.1, YP_008158817.1, WP_007032449.1, YP_004500375.1, YP_008016881.1, YP_007037692.1, NP_792775.1, AEV86039.1, WP_018784456.1, YP_003838279.1, WP_020515513.1, AEK47862.1, WP_020632413.1, YP_008090210.1, ADL46539.1, WP_020642349.1, WP_005160773.1, YP_005531112.1, YP_003422449.1, WP_022025654.1, WP_017708319.1, ETK30884.1, WP_007456997.1, YP_008736470.1, WP_020523525.1, YP_004082763.1, WP_004330614.1, WP_016325199.1, WP_009708658.1, WP_018789618.1, WP_018825055.1, WP_018805901.1, WP_018907983.1, WP_018799356.1, WP_018794911.1, WP_018805026.1, WP_018585949.1, WP_020510955.1, WP_018792999.1, WP_020217074.1, WP_018587485.1, WP_019031777.1, WP_021483737.1, WP_003426419.1, YP_003339275.1, NP_059255.1, WP_023534296.1, EQC56406.1, WP_018802752.1, YP_001649091.1, YP_004029681.1, NP_631281.1, WP_007388223.1, YP_001536372.1, WP_021455106.1, WP_019033269.1, WP_018801880.1, WP_016568267.1, BAO20175.1, WP_009191191.1, WP_003416971.1, WP_020304790.1, WP_020546322.1, YP_008719960.1, ETK34601.1, YP_004376220.1, WP_016697932.1, YP_008749003.1, AGS18777.1, YP_008191728.1, YP_003429337.1, WP_018822179.1, WP_018564505.1, XP_003284992.1, WP_018550260.1, YP_007077983.1, WP_020666466.1, WP_007445834.1, XP_642725.1, XP_644534.1, WP_003972580.1, WP_003971920.1, XP_004360524.1, WP_018100883.1, WP_018518993.1, WP_021160471.1, WP_010736568.1, WP_004933259.1, ABB05775.1, YP_003681425.1, CBJ12775.1, WP_002629300.1, WP_016699026.1, WP_018840295.1, WP_002096528.1, WP_002108160.1, ABN84819.2, ABO07336.2, YP_005709556.1, WP_002424908.1, AEB43667.1, CAH37353.1, WP_003307184.1, WP_010196438.1, WP_003339109.1, WP_016826549.1, WP_002368504.1, WP_019327859.1, WP_002367502.1, YP_005465258.1, WP_019327250.1, WP_017571417.1, WP_006132183.1, WP_019611224.1, WP_000424247.1, WP_007389494.1, NP_624952.1, WP_017605263.1, YP_005558704.1, WP_009187823.1, NP_391555.1, YP_006233542.1, AAC37123.1, EGH78566.1, YP_005563033.1, WP_010332222.1, WP_019712592.1, WP_003235751.1, YP_004879248.1, WP_017696013.1, WP_010328910.1, YP_008831837.1, WP_018843391.1, YP_004403714.1, YP_006711948.1, WP_009336793.1, WP_007073753.1, WP_016885170.1, WP_017473964.1, YP_007952701.1, WP_018837162.1, WP_004988891.1, WP_007462101.1, WP_017149289.1, WP_019854627.1, YP_004965208.1, WP_004992076.1, WP_004667654.1, WP_017329537.1, WP_005743798.1, YP_004921233.1, WP_004978811.1, WP_018851664.1, WP_010096282.1, WP_016827612.1, WP_006761996.1, WP_017684062.1, WP_005756176.1, WP_020123135.1, WP_003988453.1, YP_008794257.1, WP_016615540.1, WP_002415598.1, YP_003100261.1, WP_002390689.1, WP_019853974.1, CAJ88391.1, WP_002386225.1, WP_020646664.1, YP_289329.1, ABO21066.1, YP_004806737.1, WP_017559450.1, WP_003266960.1, YP_004811661.1, YP_002750179.1, WP_009715072.1, ADI06951.1, YP_246724.1, YP_006908538.1, WP_017539126.1, ETK35620.1, NP_845173.1, WP_002127752.1, WP_020545945.1, YP_002530382.1, WP_000742278.1, YP_002446262.1, WP_002032628.1, WP_017560162.1, WP_002165626.1, WP_000742277.1, WP_016087529.1, WP_016122671.1, WP_002185657.1, YP_008819468.1, WP_000850062.1, WP_000850060.1, WP_017586616.1, YP_006644220.1, WP_000742281.1, WP_002189869.1, WP_002012991.1, WP_002142085.1, YP_006598235.1, YP_036911.1, WP_003263770.1, WP_000850058.1, WP_001975672.1, WP_005320312.1, WP_002147340.1, WP_016101260.1, WP_000742280.1, WP_002136749.1, WP_000742283.1, YP_002338826.1, WP_016095593.1, WP_000742275.1, WP_000753781.1, NP_979159.1, WP_000753780.1, WP_000850066.1, WP_002065771.1, YP_001645457.1, YP_005572785.1, WP_000742273.1, WP_000742276.1, YP_006828834.1, YP_008780807.1, WP_002085072.1, WP_000753785.1, WP_016108573.1, WP_000753786.1, NP_832577.1, WP_021723321.1, WP_017369843.1, YP_001033450.1, WP_019299328.1, NP_268108.1, WP_004257169.1, YP_003354528.1, YP_005876829.1, YP_007931227.1, YP_007509261.1, YP_004779174.1, WP_019293977.1, WP_017865062.1, YP_007000188.1, WP_021214169.1, ABE23213.1, EUN33952.1, WP_019335881.1, YP_811776.1, YP_005869291.1, WP_019291691.1, YP_001695749.1, WP_003136475.1, YP_003102369.1, AEK39917.1, WP_007504711.1, YP_003765307.1, ACR33038.1, WP_009720339.1, WP_005739338.1, WP_005618360.1, YP_005463001.1, WP_022736320.1, WP_004928748.1, NP_826400.1, WP_004960064.1, WP_019432714.1, WP_004417574.1, CBA72693.1, WP_009299246.1, WP_021598418.1, WP_003083450.1, WP_016822997.1, WP_020667665.1, WP_019815335.1, YP_001375206.1, 3UAM, WP_005183097.1, AHH21957.1, WP_010720752.1, YP_001623972.1, WP_004918900.1, WP_017699841.1, YP_006487318.1, WP_016623271.1, WP_016091540.1, WP_010750485.1, WP_004881887.1, WP_020636677.1, WP_002315774.1, WP_002373899.1, WP_019255926.1, AHE26965.1, WP_002335193.1, WP_002310159.1, WP_002291044.1, YP_006487317.1, WP_002344007.1, YP_443713.1, WP_002345517.1, WP_010720753.1, WP_016610928.1, YP_008825128.1, YP_007916758.1, EOH54774.1, WP_009906626.1, WP_007035183.1, WP_005154978.1, WP_009077189.1, WP_018686705.1, WP_010750484.1, WP_002314077.1, WP_021599713.1, WP_020013689.1, WP_005876645.1, WP_019818585.1, WP_009717766.1, WP_010736127.1, YP_008823528.1, WP_016177717.1, WP_019722919.1, WP_005886678.1, YP_004456233.1, WP_010826815.1, YP_005319705.1, WP_010816864.1, YP_003302126.1, WP_007542878.1, YP_007154449.1, WP_010762355.1, WP_003067405.1, WP_007475873.1, WP_019818587.1, WP_010750486.1, ETK38536.1, WP_008008540.1, AHH99914.1, WP_007904886.1, WP_009049244.1, WP_007966584.1, CAK15368.1, WP_008023761.1, WP_016703840.1, AFJ58994.1, WP_009044126.1, ABA75307.1, WP_017535213.1, EUJ52442.1, ACA71302.1, WP_019817551.1, WP_003192686.1, WP_017619883.1, WP_023965598.1, WP_010458946.1, WP_023630096.1, WP_008086858.1, WP_003223871.1, WP_005789853.1, WP_007958235.1, WP_007924820.1, WP_024013398.1, WP_021477724.1, AHG21921.1, WP_009043046.1, YP_259198.1, WP_009048114.1, WP_017475573.1, WP_017139112.1, WP_010169654.1, WP_007908105.1, WP_023968628.1, WP_016987059.1, WP_010121142.1, WP_003215256.1, WP_008430815.1, WP_007920556.1, WP_010107117.1, WP_016704903.1, WP_016698261.1, WP_023582990.1, AAQ43729.1, WP_017417865.1, YP_008012282.1, ACY79422.1, WP_009315296.1, WP_006539217.1, WP_003241828.1, YP_003920455.1, YP_007186507.1, YP_003866369.1, WP_010334511.1, YP_004877529.1, WP_019258618.1, YP_008950182.1, ERE49846.1, AAR43285.1, WP_010788974.1, YP_003491304.1, WP_005486591.1, WP_020270695.1, YP_003973594.1, WP_008743992.1, WP_019811616.1, WP_023550309.1, WP_003999545.1, YP_007863677.1, YP_077759.1, WP_006536210.1, YP_008399168.1, YP_006215378.1, YP_002152363.1, YP_008076693.1, WP_009330152.1, WP_004250149.1, WP_017628514.1, WP_016265167.1, YP_008825883.1, WP_020496752.1, WP_017972707.1, WP_019856056.1, YP_395619.1, WP_019056650.1, EUJ22797.1, WP_002366886.1, WP_019611225.1, WP_020645931.1, WP_019525140.1, WP_016434950.1, WP_019065834.1, WP_020138913.1, YP_001102389.1, WP_006131607.1, WP_016696830.1, BAD01591.1, WP_018565636.1, WP_018103126.1, YP_008386612.1, WP_003990482.1, ADN98571.1, WP_018842628.1, WP_006131083.1, YP_007523944.1, CAD64126.1, ACT62315.1, WP_020123308.1, CAA55284.1, WP_004000988.1, WP_007382284.1, WP_021731441.1, WP_004931038.1, WP_019525701.1, WP_007498048.1, WP_020132274.1, CAA74695.1, WP_010037391.1, WP_005475182.1, WP_003996101.1, WP_004987638.1, WP_006130276.1, WP_020271731.1, YP_003510407.1, YP_006245475.1, YP_003494430.1, WP_009188783.1, AHF76162.1, YP_003511427.1, WP_007449889.1, NP_627062.1, WP_019325598.1, WP_021879034.1, WP_003958726.1, WP_023417290.1, YP_007745127.1, WP_018469359.1, AHF78748.1, WP_019761463.1, EUJ21901.1, WP_005919485.1, YP_003713174.1, AHF79307.1, WP_021323726.1, NP_929598.1, WP_016641617.1, WP_019993726.1, WP_002829843.1, WP_018544910.1, CBA72418.1, WP_008842437.1, ETS30755.1, YP_003041006.1, YP_004911424.1, CBA74562.1, WP_005730177.1, WP_017373446.1, YP_666953.1, YP_003380116.1, WP_006317312.1, WP_018531766.1, CBA71527.1, WP_020391472.1, ETX44942.1, WP_020554979.1, WP_023541506.1, WP_017893602.1, WP_017237123.1, CDL81819.1, WP_019434492.1, YP_001479704.1, YP_007345661.1, YP_008792493.1, YP_003511947.1, WP_016908528.1, YP_008139560.1, YP_008231509.1, CDL80158.1, CDG13442.1, YP_003466653.1, WP_018091361.1, YP_004964970.1, WP_023553690.1, WP_023530029.1, WP_003986797.1, WP_004936574.1, YP_004502087.1, WP_004873941.1, WP_019453872.1, WP_016575434.1, WP_009717599.1, BAO35063.1, ETX46865.1, WP_004722440.1, YP_004817818.1, AHG18937.1, YP_004924995.1, YP_001826252.1, WP_009324505.1, WP_018520915.1, WP_018561589.1, WP_018106210.1, AHH95463.1, WP_007264225.1, WP_006350957.1, WP_018890155.1, WP_018549773.1, WP_018381567.1, WP_020557603.1, YP_004905217.1, WP_019765912.1, WP_018958601.1, YP_004802744.1, WP_006124799.1, YP_001826219.1, YP_007931262.1, WP_010062790.1, WP_007451853.1, WP_003953200.1, WP_003953352.1, AFH95775.1, YP_004806801.1, WP_019080227.1, WP_019084365.1, WP_005317157.1, WP_020668964.1, WP_006375172.1, WP_003986796.1, WP_018518013.1, WP_018555794.1, WP_010766008.1, YP_006991088.1, WP_009741352.1, WP_010054809.1, YP_710419.1, WP_018105689.1, WP_010770878.1, WP_010760351.1, WP_002403699.1, WP_002367850.1, WP_016631105.1, NP_814154.1, WP_002370186.1, WP_002385386.1, WP_002366066.1, YP_004935323.1, WP_018640172.1, WP_010751403.1, WP_002347969.1, WP_010724889.1, WP_010728656.1, WP_010728450.1, WP_010722491.1, WP_004910963.1, WP_002322738.1, WP_019722750.1, ABW15201.1, YP_008106150.1, AFM71713.1, WP_005876331.1, WP_002324394.1, WP_002318632.1, WP_002328196.1, WP_002391772.1, WP_010744240.1, WP_002310335.1, NP_903159.1, YP_008823826.1, WP_002315302.1, WP_002341264.1, WP_002330256.1, WP_010742521.1, WP_018505439.1, WP_004240221.1, YP_007503878.1, WP_006349144.1, WP_010047974.1, ETT03311.1, ETT05353.1, WP_006659786.1, WP_018511188.1, EGH28780.1, WP_018958566.1, WP_019886636.1, EUA12966.1, WP_019764728.1, WP_018488816.1, YP_006214658.1, WP_004920061.1, WP_007457424.1, WP_008915720.1, WP_008911093.1, WP_018846405.1, WP_022643519.1, WP_009932247.1, YP_994752.1, YP_002814403.1, WP_006023856.1, WP_017949822.1, YP_003682176.1, YP_006245334.1, WP_019607819.1, WP_017563678.1, WP_017566120.1, WP_016471101.1, WP_017546664.1, WP_018851106.1, WP_018840067.1, EUA01096.1, WP_020667230.1, WP_002340311.1, YP_006882143.1, WP_019357692.1, WP_017601502.1, WP_017537444.1, WP_017559476.1, WP_017539160.1, WP_017583885.1, WP_017591883.1, WP_017606579.1, WP_017536161.1, WP_017625483.1, WP_017615731.1, WP_017610954.1, WP_010692670.1, WP_021598550.1, WP_017578006.1, WP_020657508.1, ADO70665.1, AFR09379.1, YP_007040301.1, YP_004800577.1, CAJ89556.1, WP_003979198.1, NP_624799.1, WP_007389633.1, WP_016909472.1, WP_018533752.1, WP_006601779.1, WP_019324299.1, WP_018088712.1, WP_020658520.1, YP_003683189.1, WP_019610623.1, BAA75647.1, AEM85420.1, WP_017568433.1, WP_017590176.1, WP_009076652.1, WP_017573254.1, WP_019546502.1, YP_008010297.1, YP_001824468.1, WP_017714464.1, YP_007929175.1, BAG68872.1, AGK75150.1, WP_017983178.1, BAG23684.1, WP_007451694.1, WP_007034294.1, WP_009082126.1, WP_008749364.1, ACU38929.1, WP_017626579.1, WP_004978776.1, WP_018513003.1, WP_019356311.1, WP_005164095.1, WP_018543188.1, WP_003962640.1, WP_020421329.1, WP_023536531.1, WP_018960469.1, WP_003983705.1, WP_018837857.1, WP_019766345.1, WP_018550747.1, WP_017585982.1, WP_016470980.1, WP_008409343.1, WP_009062856.1, WP_018491107.1, WP_017547044.1, WP_017582301.1, WP_016640753.1, WP_010060695.1, WP_018956423.1, WP_007458431.1, WP_007829986.1, WP_023538786.1, WP_018515122.1, AGO05942.1, YP_003765663.1, 2BEN, WP_003103314.1, YP_007952963.1, ADP65784.1, AFU00858.1, EUJ29711.1, WP_003108699.1, WP_018551448.1, YP_008789894.1, WP_020633914.1, WP_016082979.1, YP_008794881.1, WP_007455317.1, YP_006878156.1, BAE74790.1, YP_008597425.1, CBA73223.1, WP_010223097.1, WP_004254296.1, YP_003767975.1, WP_023507720.1, WP_003058636.1, YP_005390472.1, YP_005414804.1, EFH99651.1, WP_007639635.1, WP_005897810.1, WP_002341204.1, WP_019524606.1, YP_005390769.1, 1EXG, WP_004173205.1, WP_008580855.1, ACR47067.1, AFD19665.1, AFC74836.1, AFB26282.1, WP_016538814.1, ETK35382.1, WP_016945690.1, WP_016926269.1, WP_017626505.1, YP_004885020.1, YP_008893727.1, WP_023714616.1, YP_005295675.1, YP_001495062.1, YP_005301048.1, YP_001650335.1, YP_005426704.1, WP_016916801.1, YP_002916114.1, YP_005392896.1, YP_005426397.1, YP_005390473.1, WP_016728195.1, YP_005302195.1, WP_023160944.1, CBA72266.1, WP_023752142.1, EUJ25258.1, YP_005365725.1, EUJ39020.1, WP_010423051.1, WP_010223096.1, WP_008580442.1

**GenBank accession numbers of protein sequences included in AA11exp.**

WP_019930921.1, XP_005157085.1, XP_005157086.1, XP_687774.6, XP_004025562.1, XP_004951014.1, XP_005061457.1, EJK60153.1, XP_005430215.1, YP_001687010.1, XP_004176585.1, WP_007088891.1, XP_002490708.1, XP_001217846.1, XP_765530.1, XP_002405770.1, YP_007950446.1, XP_001616256.1, XP_002850634.1, XP_002291264.1, XP_003287028.1, XP_001119962.2, CBK24481.2, EPB86587.1, CAF99582.1, BAF74050.1, AEQ01053.1, WP_021461970.1, AAA29730.1, BAH20667.1, GAA86711.1, EHA27022.1, CAH10285.1, YP_004420294.1, EGZ30744.1, YP_002775203.1, EPR57290.1, ESS33425.1, AAD12605.1, XP_004535664.1, XP_003341942.1, ELQ35674.1, XP_002599930.1, XP_006475459.1, EFZ00747.1, XP_005804023.1, XP_006475460.1, CCC14087.1, XP_005834078.1, XP_002838570.1, XP_003836919.1, EFX73172.1, XP_001508877.2, XP_006451478.1, BAA34309.1, EQB58862.1, XP_001258215.1, ELA32984.1, EKC22279.1, WP_005694766.1, ELQ60037.1, ELQ36562.1, XP_003880553.1, EON98853.1, XP_002147803.1, XP_003716281.1, XP_002720147.1, XP_003716280.1, WP_022342943.1, EFY91408.1, YP_004385539.1, ESK87909.1, XP_001797832.1, XP_005767260.1, ABK57030.1, AAF73803.1, EME45679.1, WP_018346332.1, XP_002614066.1, XP_001850961.1, WP_018075661.1, WP_002639051.1, WP_021009666.1, CDJ46794.1, XP_750980.1, XP_003305986.1, ESP00338.1, ENH82340.1, XP_003349120.1, ESK87157.1, ETI21120.1, XP_001936380.1, XP_002741718.1, XP_002540653.1, EGU87901.1, XP_006236751.1, XP_006236752.1, XP_006225023.1, XP_006225022.1, XP_002114296.1, AAL90445.1, AAG16729.1, WP_008565793.1, ETN41984.1, AAF73782.1, XP_958127.1, WP_023396525.1, WP_023396848.1, AAF73821.1, AAF73826.1, XP_001242938.1, XP_003352667.1, WP_000458114.1, ELQ61523.1, XP_006225024.1, XP_006670400.1, EGZ67778.1, XP_964365.1, XP_006236753.1, XP_003843789.1, EGZ76857.1, CAK45829.1, AAF73774.1, WP_001842818.1, EHK43392.1, YP_007815470.1, EGO52047.1, EHY59273.1, EMC94923.1, WP_000477090.1, EKG21662.1, BAK01092.1, WP_000477112.1, XP_001906381.1, XP_001590143.1, WP_000477106.1, ETS78924.1, EOA83923.1, AAF73783.1, AAF73793.1, AAF73794.1, CCD48690.1, WP_000477109.1, EMD84831.1, ETS05308.1, EMD65813.1, XP_003307044.1, WP_007464073.1, WP_001837074.1, ESZ97766.1, XP_006673354.1, EMD85633.1, XP_002556576.1, EKG12748.1, EGO54744.1, XP_005775219.1, XP_002674179.1, AFD97213.1, EOO01866.1, WP_001812308.1, EMS24852.1, EFZ04469.1, ELQ36644.1, XP_002623110.1, EFY91792.1, XP_005776160.1, EFZ18402.1, XP_001268194.1, ESU05849.1, XP_003053715.1, AAF73789.1, XP_963492.1, EKJ75109.1, XP_001801474.1, WP_001836749.1, EKD14716.1, ETI24548.1, XP_001941363.1, EJP63131.1, EJT71157.1, CCU83020.1, EPQ64850.1, EPS44930.1, ELQ68311.1, ENH61451.1, EMT61439.1, EPE10862.1, EGU83897.1, XP_001550507.1, EGX47272.1, EGO56821.1, YP_005072077.1, XP_001792664.1, XP_003033631.1, XP_380818.1, XP_003346716.1, YP_007092178.1, EFZ01840.1, EME42943.1, EMT62061.1, ENH80220.1, XP_002792749.1, EKM81819.1, EJT69668.1, EEH49643.1, ETN44638.1, EFY86242.1, XP_001276229.1, XP_001220488.1, EGX43927.1, EME82432.1, EFX02550.1, EMF11929.1, EFZ01910.1, ETI56552.1, ETN24563.1, YP_004200558.1, EMR68381.1, XP_002835569.1, EON64769.1, ETS04461.1, EQB52923.1, WP_004628701.1, EUC47694.1, YP_007099258.1, EOA81334.1, CCX07292.1, YP_003372628.1, CCF32152.1, XP_003025876.1, EMD65510.1, WP_019362746.1, EUC38776.1, XP_774319.1, XP_571909.1, EIW68339.1, XP_002567027.1, XP_003709778.1, CCT62801.1, XP_003850030.1, XP_001597818.1, EGY22619.1, EMS21926.1, EUN31488.1, EGX53029.1, AHF20627.1, AFD97212.1, AFD97209.1, AFD97206.1, AFD97205.1, AFD97214.1, AFD97211.1, AFD97210.1, EMC93960.1, EKG12761.1, CCX14970.1, XP_003174002.1, XP_006692077.1, EMD67171.1, ENH82730.1, XP_002382772.1, XP_001228385.1, BAE61530.1, CCE27776.1, EPS39968.1, ELR07584.1, EOD46824.1, CAO91829.1, XP_006668451.1, XP_003007312.1, EEH23200.1, EFQ32945.1, XP_002544405.1, EOA84296.1, EGY17657.1, XP_003303971.1, CBF74037.1, EUC44793.1, XP_001266231.1, XP_003667236.1, XP_003006162.1, XP_003235451.1, EDP51150.1, XP_748042.1, EIT79819.1, AFR96628.1, ERS99096.1, XP_002789213.1, EGD92942.1, XP_001937368.1, EGE04098.1, EJT82590.1, EUC32448.1, EUN29191.1, CCF45557.1, ERS94950.1, XP_002569065.1, XP_003658112.1, YP_006065316.1, XP_003019976.1, XP_003015133.1, XP_002848955.1, XP_960937.1, EFQ36410.1, XP_001909751.1, AFD97208.1, EGC46978.1, ELA36568.1, EMR83340.1, CCD52974.1, CCD54857.1, EEH05325.1, XP_001547004.1, XP_002481851.1, EMD86119.1, XP_001549934.1, XP_002840711.1, XP_001217899.1, GAA88290.1, XP_001543425.1, ETS76855.1, CCT69101.1, XP_001393828.1, EME87736.1, XP_386263.1, XP_001242606.1, CCX31247.1, ELR02541.1, XP_003069792.1, EUC45496.1, EHK42112.1, XP_001791303.1, EJT74193.1, XP_003712585.1, XP_002842272.1, XP_002127986.1, GAD93568.1, XP_003660925.1, EUC37748.1, EHY55011.1, ESS55205.1, AHF20644.1, EKD00592.1, EMT68300.1, EFQ29865.1, ENH68621.1, CCT67099.1, ELQ43122.1, EKJ75600.1, ESZ94362.1, EPE24813.1, XP_003843057.1, EJP64324.1, XP_003297367.1, EKV17912.1, EFW99073.1, EPS34477.1, EKG20672.1, ETS75570.1, ETS77577.1, ELA24851.1, ENH83182.1, EOA82325.1, EKV15847.1, CBY24786.1, XP_003305261.1, EGU85465.1, EOD53021.1, EUN29326.1, XP_001931006.1, XP_003437316.1, XP_002568660.1, XP_001594978.1, XP_002147680.1, ENH74483.1, EHK49951.1, ESZ98276.1, EME49105.1, EMD58702.1, ENI08410.1, XP_001822663.2, EMR67112.1, GAA98995.1, ACZ65015.1, EGO60701.1, EHK17484.1, EKG19379.1, EON97679.1, EKD13625.1, EEQ90950.1, EGC44079.1, EKV10928.1, XP_001214248.1, CCF40911.1, EQL33487.1, EMR68901.1, XP_002628516.1, ETS77073.1, EKG09287.1, EHA52406.1, XP_003348222.1, EKJ77892.1, XP_382598.1, EGE82146.1, EME82188.1, EMD61301.1, EGE05564.1, XP_001227363.1, EFW18701.1, ESA14427.1, EJP65660.1, EGU76897.1, XP_001940795.1, EKG12228.1, EON63048.1, EKG18934.1, XP_003045352.1, EGR49412.1, EEH50753.1, EUC44582.1, YP_007764325.1, EUN22101.1, EUC30645.1, XP_001396774.1, EHK19854.1, XP_003836245.1, EMR69119.1, XP_003650198.1, EMD86748.1, EHK16770.1, YP_001508531.1, XP_003169269.1, GAA90553.1, EDP49665.1, EME43051.1, EOA85398.1, XP_003850477.1, CCX34923.1, EQB58330.1, ERF68296.1, EEH15819.1, XP_002153103.1, XP_001243592.1, EMR69820.1, AFG25472.1, ADN94504.1, ADN94506.1, AAR05777.2, XP_748715.1, ENH71945.1, EGR50937.1, XP_384016.1, EMD94762.1, XP_003708917.1, EJT51965.1, ETS02825.1, XP_002842832.1, EOD43831.1, XP_001797251.1, EMT70525.1, EGY21943.1, XP_003050482.1, ESU06251.1, XP_002837977.1, CCX07293.1, CCU74383.1, ERF75887.1, EPQ66731.1, CCT75452.1, CCE28971.1, ELR06576.1, EMD66052.1, EOD47246.1, EKD12205.1, EMT69478.1, EPE03832.1, EMD89928.1, GAD93338.1, XP_001393259.2, XP_002487567.1, XP_658206.1, XP_001821065.1, XP_003658966.1, XP_003029539.1, XP_001225335.1, EMD85627.1, EOA90850.1, EER38578.1, EEH06906.1, EMD84834.1, EUN20877.1, EOA83921.1, ADK37848.1, XP_004463751.1, WP_018296998.1, EKJ73219.1, EEH09607.1, EGC44326.1, ESU09345.1, XP_002566688.1, ELA28398.1, XP_001548564.1, EMR90530.1, YP_001504935.1, XP_001593829.1, EON61299.1, EON62244.1, XP_003235677.1, XP_002340103.1, XP_003069064.1, EGS21088.1, EMD66417.1, CCD44596.1, EHA24588.1, EER38195.1, BAE66259.1, EIT75494.1, EUC40825.1, XP_003032165.1, GAA94037.1, XP_003837919.1, XP_003712361.1, XP_001225174.1, CAQ16288.1, XP_005788994.1, XP_003710267.1, CCX07996.1, ELQ59175.1, XP_003713232.1, XP_003029201.1, XP_003047765.1, XP_001911580.1, XP_002838243.1, EMF10416.1, CCE28972.1, XP_003050224.1, XP_003026574.1, CCE32966.1, XP_003024865.1, EOA85932.1, EMF11619.1, EMF17657.1, EHK97268.1, EEH09854.1, EEQ89326.1, ETN47196.1, XP_003649387.1, EQB46896.1, ENH99059.1, XP_002835201.1, CCX34395.1, WP_000641475.1, XP_001542256.1, ETS82466.1, EKD20942.1, EGD98498.1, EFY88319.1, XP_003969965.1, ELA24573.1, EON65846.1, XP_003195343.1, XP_003649083.1, XP_001544052.1, XP_002621360.1, EFZ02283.1, WP_001820835.1, ETI41793.1, ETK81841.1, ETM41733.1, ETL35250.1, ETP39665.1, XP_003856632.1, GAD94262.1, EGE80900.1, EEH16034.1, EEH50505.1, EER37945.1, EME45691.1, EQL04114.1, EGD99938.1, ELQ43590.1, EMD01144.1, EQL37881.1, EEQ89863.1, XP_001912736.1, EOA90842.1, EJT76146.1, XP_006225025.1, EGR51219.1, EUN21907.1, XP_003013767.1, XP_002622207.1, EGE85732.1, EEQ89606.1, GAA95388.1, EPE05022.1, CCF33133.1, XP_002563773.1, XP_003661558.1, EQB56592.1, XP_002562514.1, XP_002621567.1, XP_002566969.1, XP_002556886.1, EEQ83434.1, XP_003029948.1, GAA93289.1, EMD86612.1, WP_019251310.1, WP_019253884.1, EUC40374.1, CCX07291.1, EMT68301.1, EMD66420.1, EMD65810.1, YP_004648833.1, XP_001228963.1, XP_001541987.1, XP_003026131.1, YP_008326748.1, EPE26388.1, ADX31294.1, XP_002842573.1, XP_003231393.1, EGE06977.1, XP_003171247.1, XP_003011652.1, XP_003656955.1, EME84178.1, XP_002789291.1, XP_003016696.1, XP_001823731.1, XP_002380776.1, EHK20062.1, XP_002566537.1, XP_003003787.1, EMC92258.1, EPS45954.1, ELR08410.1, CAP73493.1, EUC26810.1, XP_003070051.1, EAS31685.2, XP_002566575.1, XP_001259157.1, XP_001273546.1, XP_002543707.1, XP_005761324.1, XP_002482903.1, EQL00501.1, EHK43388.1, XP_003843790.1, EKG19378.1, EJP67667.1, XP_003174561.1, XP_003236308.1, XP_003018368.1, EGE03508.1, XP_002847024.1, EOD47354.1, EHK21327.1, XP_001212055.1, CCT62295.1, EFY85839.1, EOD49695.1, XP_006667385.1, XP_003854534.1, EFY95565.1, XP_002839220.1, EGD93178.1, ERT01344.1, XP_006673865.1, EQL01822.1, XP_003174226.1, XP_003231518.1, EJP67690.1, EGE03336.1, EME49904.1, EKG09975.1, EMR61516.1, XP_003021860.1, EFY94360.1, EGE09367.1, EMF16505.1, XP_003856402.1, XP_002841287.1, EFY97908.1, XP_001827392.2, XP_002384586.1, EME87785.1, BAN38453.1, WP_003664232.1
